# Protein-altering germline mutations implicate novel genes related to lung cancer development

**DOI:** 10.1038/s41467-020-15905-6

**Published:** 2020-05-11

**Authors:** Xuemei Ji, Semanti Mukherjee, Maria Teresa Landi, Yohan Bosse, Philippe Joubert, Dakai Zhu, Ivan Gorlov, Xiangjun Xiao, Younghun Han, Olga Gorlova, Rayjean J. Hung, Yonathan Brhane, Robert Carreras-Torres, David C. Christiani, Neil Caporaso, Mattias Johansson, Geoffrey Liu, Stig E. Bojesen, Loic Le Marchand, Demetrios Albanes, Heike Bickeböller, Melinda C. Aldrich, William S. Bush, Adonina Tardon, Gad Rennert, Chu Chen, Jinyoung Byun, Konstantin H. Dragnev, John K. Field, Lambertus FA. Kiemeney, Philip Lazarus, Shan Zienolddiny, Stephen Lam, Matthew B. Schabath, Angeline S. Andrew, Pier A. Bertazzi, Angela C. Pesatori, Nancy Diao, Li Su, Lei Song, Ruyang Zhang, Natasha Leighl, Jakob S. Johansen, Anders Mellemgaard, Walid Saliba, Christopher Haiman, Lynne Wilkens, Ana Fernandez-Somoano, Guillermo Fernandez-Tardon, Erik H. F. M. van der Heijden, Jin Hee Kim, Michael P. A. Davies, Michael W. Marcus, Hans Brunnström, Jonas Manjer, Olle Melander, David C. Muller, Kim Overvad, Antonia Trichopoulou, Rosario Tumino, Gary E. Goodman, Angela Cox, Fiona Taylor, Penella Woll, Erich Wichmann, Thomas Muley, Angela Risch, Albert Rosenberger, Kjell Grankvist, Mikael Johansson, Frances Shepherd, Ming-Sound Tsao, Susanne M. Arnold, Eric B. Haura, Ciprian Bolca, Ivana Holcatova, Vladimir Janout, Milica Kontic, Jolanta Lissowska, Anush Mukeria, Simona Ognjanovic, Tadeusz M. Orlowski, Ghislaine Scelo, Beata Swiatkowska, David Zaridze, Per Bakke, Vidar Skaug, Lesley M. Butler, Kenneth Offit, Preethi Srinivasan, Chaitanya Bandlamudi, Matthew D. Hellmann, David B. Solit, Mark E. Robson, Charles M. Rudin, Zsofia K. Stadler, Barry S. Taylor, Michael F. Berger, Richard Houlston, John McLaughlin, Victoria Stevens, David C. Nickle, Ma’en Obeidat, Wim Timens, María Soler Artigas, Sanjay Shete, Hermann Brenner, Stephen Chanock, Paul Brennan, James D. McKay, Christopher I. Amos

**Affiliations:** 1 Biomedical Data Science, Geisel School of Medicine at Dartmouth, Hanover, NH USA; 2 Department of Medicine, Memorial Sloan Kettering Cancer Center, New York, NY USA; 3 Division of Cancer Epidemiology and Genetics, National Cancer Institute, National Institutes of Health, Bethesda, MD USA; 4 Institut universitaire de cardiologie et de pneumologie de Québec, Department of Molecular Medicine, Laval University, Québec, Canada; 5 The Institute for Clinical and Translational Research, Baylor College of Medicine, Houston, TX USA; 6 Lunenfeld-Tanenbaum Research Institute, Sinai Health System and University of Toronto, Toronto, Canada; 7 International Agency for Research on Cancer, World Health Organization, Lyon, France; 8 Department of Environmental Health, Harvard T.H. Chan School of Public Health, Boston, MA USA; 9 Department of Medicine, Massachusetts General Hospital/Harvard, Boston, MA USA; 10 Department of Clinical Biochemistry, Herlev and Gentofte Hospital, Copenhagen University Hospital, Copenhagen, Denmark; 11 Faculty of Health and Medical Sciences, University of Copenhagen, Copenhagen, Denmark; 12 Copenhagen General Population Study, Herlev and Gentofte Hospital, Copenhagen, Denmark; 13 Epidemiology Program, University of Hawaii Cancer Center, Honolulu, HI USA; 14 Department of Genetic Epidemiology, University Medical Center, Georg-August-University Göttingen, Göttingen, Germany; 15 Department of Thoracic Surgery, Division of Epidemiology, Vanderbilt University Medical Center, Göttingen, Germany; 16 Department of Epidemiology and Biostatistics, School of Medicine, Case Western Reserve University, Cleveland, OH USA; 17 IUOPA. University of Oviedo and CIBERESP, Faculty of Medicine, Campus del Cristo s/n, Oviedo, Spain; 18 Clalit National Cancer Control Center at Carmel Medical Center and Technion Faculty of Medicine, Haifa, Israel; 19 Public Health Sciences Division, Fred Hutchinson Cancer Research Center, Seattle, WA USA; 20 The Norris Cotton Cancer Center, Dartmouth-Hitchcock Medical Center, Lebanon, NH USA; 21 Roy Castle lung Cancer Research Programme, Institute of Translational Medicine, University of Liverpool, Liverpool, United Kingdom; 22 Radboud University Medical Center, Radboud Institute for Health Sciences, Nijmegen, The Netherlands; 23 Department of Pharmaceutical Sciences, College of Pharmacy, Washington State University, Spokane, WA USA; 24 National Institute of Occupational Health, Oslo, Norway; 25 British Columbia Cancer Agency, Vancouver, Canada; 26 Department of Cancer Epidemiology, H. Lee Moffitt Cancer Center and Research Institute, Tampa, FL USA; 27 Department of Epidemiology, Geisel School of Medicine, Hanover, NH USA; 28 Department of Preventive Medicine, IRCCS Foundation Ca’ Granda Ospedale Maggiore Policlinico, Milan, Italy; 29 Department of Clinical Sciences and Community Health, University of Milan, Milan, Italy; 30 University Health Network- The Princess Margaret Cancer Centre, Toronto, CA USA; 31 Department of Oncology, Herlev and Gentofte Hospital, Copenhagen University Hospital, Copenhagen, Denmark; 32 Department of Preventive Medicine, Keck School of Medicine, University of Southern California Norris Comprehensive Cancer Center, Los Angeles, CA USA; 33 Department of Integrative Bioscience & Biotechnology, Sejong University, Gwangjin-gu, Seoul Republic of Korea; 34 Department of Pathology, Lund University, Lund, Sweden; 35 Faculty of Medicine, Lund University, Lund, Sweden; 36 School of Public Health, St Mary’s Campus, Imperial College London, London, UK; 37 Hellenic Health Foundation, Athens, GR Greece; 38 Cancer Registry and Histopathology Department, “Civic - M.P. Arezzo” Hospital, Asp Ragusa, Italy; 39 Fred Hutchinson Cancer Research Center, Seattle, WA USA; 40 Swedish Medical Group, Seattle, WA USA; 41 Department of Oncology and Metabolism, University of Sheffield, Sheffield, UK; 42 Research Unit of Molecular Epidemiology, Institute of Epidemiology II, Helmholtz Zentrum München, German Research Center for Environmental Health, Neuherberg, Germany; 43 Thoraxklinik at University Hospital Heidelberg, Heidelberg, Germany; 44 Translational Lung Research Center Heidelberg (TLRC-H), Heidelberg, Germany; 45 University of Salzburg and Cancer Cluster Salzburg, Salzburg, Austria; 46 Department of Medical Biosciences, Umeå University, Umeå, Sweden; 47 Department of Radiation Sciences, Umeå University, Umeå, Sweden; 48 Princess Margaret Cancer Centre, Toronto, Canada; 49 University of Kentucky, Markey Cancer Center, Lexington, KY USA; 50 Department of Thoracic Oncology, H. Lee Moffitt Cancer Center and Research Institute, Tampa, FL USA; 51 Institute of Pneumology “Marius Nasta”, Bucharest, Romania; 52 Charles University, 1st Faculty of Medicine, Prague, Czech Republic; 53 Faculty of Health Sciences, Palacky University, Olomouc, Czech Republic; 54 Clinical Center of Serbia, Clinic for Pulmonology, School of Medicine, University of Belgrade, Belgrade, Serbia; 55 Department of Cancer Epidemiology and Prevention, M. Sklodowska-Curie Institute – Oncology Center, Warsaw, Poland; 56 Department of Epidemiology and Prevention, Russian N.N.Blokhin Cancer Research Centre, Moscow, Russian Federation; 57 International Organization for Cancer Prevention and Research, Belgrade, Serbia; 58 Department of Surgery, National Tuberculosis and Lung Diseases Research Institute, Warsaw, Poland; 59 Nofer Institute of Occupational Medicine, Department of Environmental Epidemiology, Lodz, Poland; 60 Department of Clinical Science, University of Bergen, Bergen, Norway; 61 University of Pittsburgh Cancer Institute, Pittsburgh, USA; 62 Department of Pathology, Memorial Sloan Kettering Cancer Center, New York, USA; 63 Marie-Josée and Henry R. KravisCenter for Molecular Oncology, Memorial Sloan Kettering Cancer Center, New York, USA; 64 Department of Epidemiology and Biostatistics, Memorial Sloan Kettering Cancer Center, New York, USA; 65 The Institute of Cancer Research, London, UK; 66 University of Toronto, Toronto, Canada; 67 American Cancer Society, Inc., Atlanta, GA USA; 68 Merck Research Laboratories, Genetics and Pharmacogenomics, Boston, MA USA; 69 The University of British Columbia Centre for Heart Lung Innovation, St Paul’s Hospital, Vancouver, BC Canada; 70 University of Groningen, University Medical Center Groningen, Department of Pathology and Medical Biology, GRIAC research institute, Groningen, The Netherlands; 71 Genetic Epidemiology Group, Department of Health Sciences, University of Leicester, Leicester, LE1 7RH UK; 72 National Institute for Health Research (NIHR) Leicester Respiratory Biomedical Research Unit, Glenfield Hospital, Leicester, UK; 73 Department of Biostatistics, The University of Texas MD Anderson Cancer Center, Houston, TX USA; 74 Division of Clinical Epidemiology and Aging Research, German Cancer Research Center (DKFZ), Heidelberg, Germany; 75 Dan L Duncan Comprehensive Cancer Center, 7200 Cambridge St., 7th Floor, Houston, TX 77030 USA

**Keywords:** Cancer, Computational biology and bioinformatics, Genetics

## Abstract

Few germline mutations are known to affect lung cancer risk. We performed analyses of rare variants from 39,146 individuals of European ancestry and investigated gene expression levels in 7,773 samples. We find a large-effect association with an *ATM* L2307F (rs56009889) mutation in adenocarcinoma for discovery (adjusted Odds Ratio = 8.82, *P* = 1.18 × 10^−15^) and replication (adjusted OR = 2.93, *P* = 2.22 × 10^−3^) that is more pronounced in females (adjusted OR = 6.81 and 3.19 and for discovery and replication). We observe an excess loss of heterozygosity in lung tumors among *ATM* L2307F allele carriers. L2307F is more frequent (4%) among Ashkenazi Jewish populations. We also observe an association in discovery (adjusted OR = 2.61, *P* = 7.98 × 10^−22^) and replication datasets (adjusted OR = 1.55, *P* = 0.06) with a loss-of-function mutation, Q4X (rs150665432) of an uncharacterized gene, *KIAA0930*. Our findings implicate germline genetic variants in *ATM* with lung cancer susceptibility and suggest *KIAA0930* as a novel candidate gene for lung cancer risk.

## Abstract (web summary)

In lung cancer, relatively few germline mutations are known to impact risk. Here the authors looked at rare variants in 39,146 individuals and find novel germline mutations associated with risk, as well as implicating ATM and a new candidate gene for lung cancer risk.

## Introduction

Lung cancer is a leading cause of cancer death in the U.S. and worldwide and represents a major public health problem^1^. Hereditary factors play a crucial role in lung cancer pathogenesis^2^. The first wave of genome-wide association studies^3^ identified susceptibility regions and common variants for lung cancer risk but have been restricted to analysis of more common variants having allele frequencies of 1% or higher. Few previous studies identified rare germline mutations responsible for lung cancer etiology because this type of research requires large-sample sizes and extensive genetic analysis. Although <1% of most populations are carriers of a germline mutation that drives cancer, such mutations can confer as much as an 80% lifetime risk for developing cancer^4^ and influence between 3 and 10% of cancers diagnosed yearly^5^. In addition, identification of cancer-related mutations has provided potential targets for cancer treatment and drug development. For example, the rare inherited T790M mutation of *EGFR* is associated with greatly increased risk for lung cancer in nonsmokers^6^. Individuals with this mutation do not respond well to first-generation EGFR therapy^7^ and a targeted approach is required for individuals carrying this mutation^8^. Similarly, identification of germline mutations in *BRCA1* and *BRCA2* led to the successful application of PARP inhibition therapy for breast and ovarian cancer. Defining germline mutations for lung cancer may also assist in early detection and targeted prevention, similar to the benefit conveyed in screening for deleterious *BRCA1* and *BRCA2* germline mutations^9,10^.

The objective of this study was to identify reliable germline mutations that highly affect lung cancer risk and to discover novel genes that are involved in the etiology and development of lung cancer. We analyzed two independent datasets comprising 39,146 individuals of European ancestry that have not been used previously for identifying low minor allele frequency (MAF) variants occurring in <1% of the population to discover and verify mutations having large effects increasing lung cancer risk. We confirmed the genotyping fidelity of the selected germline mutations in both datasets by repeatedly genotyping of 5,742 subjects of the discovery and replication phases and by comparing the MAFs in unaffected individuals of our both datasets to those in publically available datasets. In addition, in order to investigate the significance and properties of our discovered mutations, we analyzed the data in stratified subgroups and ethno-geographic populations, evaluated biallelic two-hit events in an additional dataset of European ancestry for available variants and performed structure-based investigation of mutations to evaluate their pathogenicity. We also explored whether the discovered mutation-related isoforms are expressed in lung by using RNA-seq data and isoform expression data, and elucidated the role of the novel mutation-related genes in lung cancer pathogenesis by studying gene expression data. Altogether, our study identified and validated two novel mutations in genes that significantly affect lung cancer etiology, offering insights for understanding lung cancer mechanisms. Our findings may provide insights for targeted lung cancer screening and drug development.

## Results

### Discovery of driver germline mutations with large effect

We used the availability of two datasets, of which 5742 individuals were genotyped on both platforms, to investigate germ-line mutations with large effects on lung cancer risk. We removed the overlapping 5742 individuals from the first case-control dataset and used it as the discovery dataset. This dataset comprised the 28,878 individuals of European ancestry genotyped on the Illumina Oncoarray. The second dataset, which consisted of 10,268 independent individuals of European ancestry genotyped on an Affymetrix Axiome genotyping array, which was used as the replication cohort. These data have also not been included in prior studies. We used the discovery dataset to identify potential rare variants linked with lung cancer susceptibility. The replication cohort was used for two validation aims, first the technical validation by considering genotyping concordance between platforms and second as an independent validation cohort for the associations identified in the discovery cohort. Results from the validation cohort have not been previously published except for a targeted analysis of SNPs near hTERT on chromosome 5q^11^. The study design is presented in Supplementary Fig. 1.

In the discovery series, to explore germline mutations conveying a substantial increase on lung cancer risk, we performed association analyses for mutations having MAF <0.01, by using the discovery dataset (Supplementary Table 1). Three mutations within the exome, including rs56009889, rs150665432, and rs61816761 had association with *P* values of <5.0 × 10^−8^ and OR values of more than 2.0 in the discovery dataset when crude association analysis were performed (Supplementary Table 2). We then validated these findings in an independent non-overlapping case-control dataset consisting of 10,268 individuals of European ancestry.

Because the samples for the discovery and replication phase of this analysis utilized different platforms, we were able to evaluate the fidelity of the arrays by studying the 5742 individuals who were genotyped using both genotyping platforms. The variants rs56009889 and rs150665432 had excellent concordances of 99.95% and 99.08% for overall genotypes and 89.66% and 92.31% for the rare alleles, respectively, which confirmed their genotyping fidelity in both datasets. However, genotyping for rs61816761 showed poor concordance; this mutation was dropped from further analysis (Supplementary Table 3). Additionally, among unaffected individuals, the Minor Allele Frequencies (MAFs) of rs56009889 and rs150665432 in both datasets were comparable to the MAFs found for European populations in the Exome Aggregation Consortium^12^, and were in agreement with MAFs in the NHLBI GO Exome Sequencing Project and the Trans-Omics for Precision Medicine Program (https://www.ncbi.nlm.nih.gov/projects/SNP/snp_ref.cgi?rs=150665432, https://www.ncbi.nlm.nih.gov/projects/SNP/snp_ref.cgi?rs=56009889) (Supplementary Table 4), which supported the reliability of the genotyping data of rs56009889 and rs150665432 in both datasets.

In the independent replication series, rs56009889 (*P* = 0.34) did not significantly associate with overall lung cancer risk without adjustment (Table 1). However, strong evidence for association was noted in the replication data for lung adenocarcinoma (LUAD) (*P* = 9.8 × 10^−4^) and women (*P* = 0.01) for rs56009889. Results for lung cancer overall are less significant after PC adjustment, but there is still a highly significant result for the *ATM*−2307F association in LUAD (*P* = 1.18 × 10^−15^ for discovery and *P* = 2.22 × 10^−3^ for replication (Table 1). The variant rs150665432 showed a significant trend (*P* = 0.06) in the much smaller replication dataset.

**Table 1:** Allele-specific lung cancer risk for ATM-L2307F (rs56009889) and KIAA0930-Q4X (rs150665432).

Mutation	Outcome	Population	Dataset	Crude		Adjust by PCs^a^	
				OR (95% CI)	*P*	OR (95% CI)	*P*
rs56009889	Lung Cancer	All	Discovery	3.98 (2.40–6.61)	8.21E−09	3.15 (1.89–5.26)	1.04E−05
			Replication	1.39 (0.70–2.73)	0.34	1.36 (0.69–2.68)	0.37
rs56009889	Lung Cancer	Female	Discovery	9.45 (3.39–26.34)	1.49E−07	6.81 (2.42–19.17)	0.0003
			Replication	3.36 (1.22–9.26)	0.01	3.19 (1.16–8.82)	0.03
rs56009889	LAD	All	Discovery	8.15 (4.86–13.68)	2.74E−21	8.82 (5.18–15.03)	1.18E−15
			Replication	3.00 (1.51–5.96)	9.76E−04	2.93 (1.47–5.82)	2.22E−03
rs150665432	Lung Cancer	All	Discovery	2.78 (2.27–3.39)	1.71E−25	2.61 (2.15–3.18)	7.98E−22
			Replication	1.54 (0.98–2.42)	0.06	1.55 (0.98–2.44)	0.06

OR, 95% CI and P values were generated from logistic regression model.

^a^PCs are the principal components.

### Histology and gender-specific lung cancer risk of *ATM*-L2307F

**Fig. 1:**
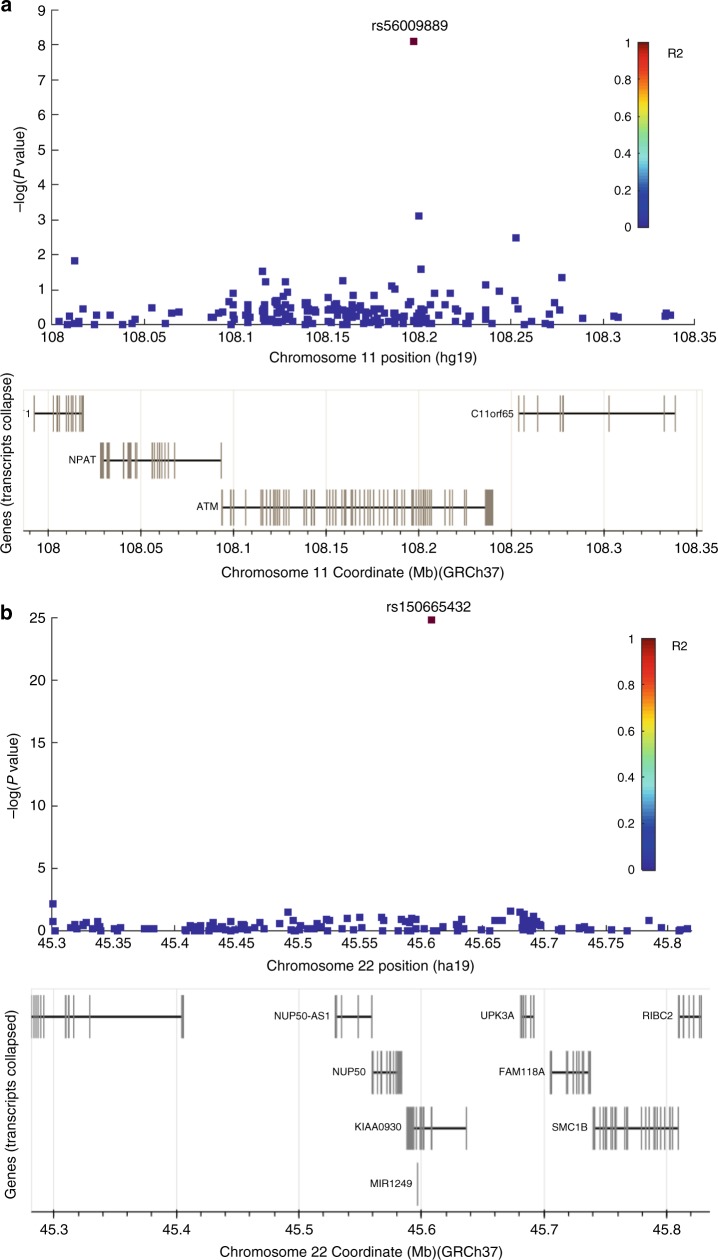
Regional lung cancer association plots for the *ATM* and *KIAA0930* risk loci. **a** *ATM* region for lung cancer risk. rs56009889, localizing to chromosome 11 and mapping within *ATM*, is not in linkage disequilibrium (LD) with any SNPs that have been identified before; **b** *KIAA0930* region for lung cancer risk. rs150665432 localizes to chromosome 22 and maps within uncharacterized *KIAA0930*, which is not in LD with any SNPs that have been identified before. For each plot, −log_10_ *P* values (y-axis) of the SNPs are shown according to their chromosomal positions (*x*-axis). The top genotyped SNP in each analysis is labeled by its rs number. The color intensity of each symbol reflects the extent of LD with the top lung cancer-associated SNP in the discovery data: blue (*r*^2^ = 0) through to red (*r*^2^ = 1.0). Physical positions are based on NCBI build 37 of the human genome. The relative positions of genes are also shown. Source data are provided as a Source Data file (Source Data 1).

**Fig. 2:**
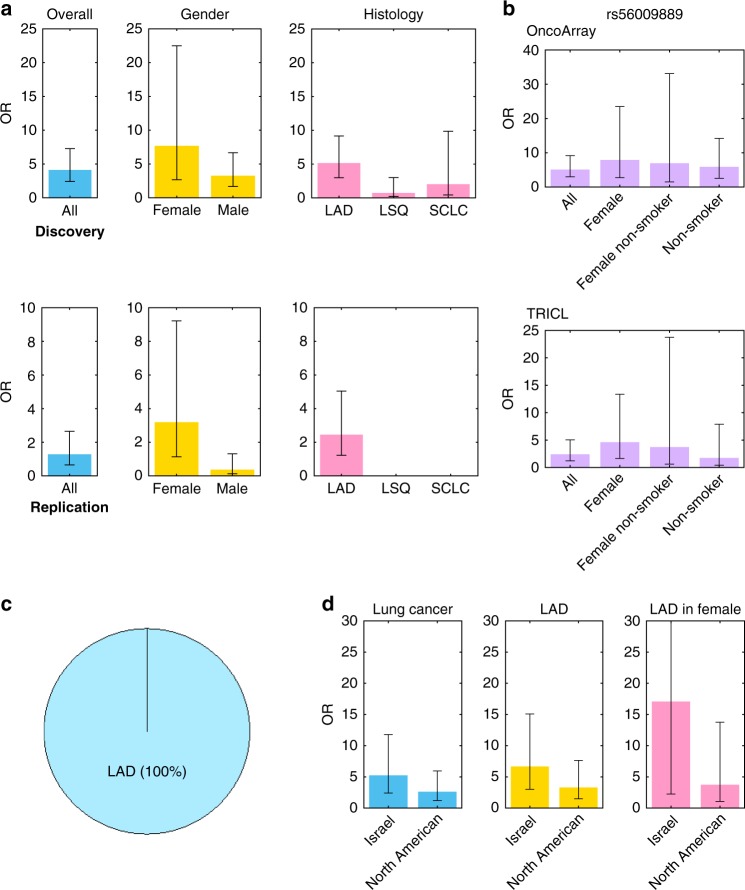
*ATM* rs56009889 association with lung cancer risk. *P* values were determined by logistic regression analysis adjusted by age, gender, smoking status and the principal components. **a** Stratified analyses of the association between rs56009889 and Lung cancer. Compared to non-carriers, L2307F carriers had an increased risk of lung cancer with ORs being 4.19 in the discovery data (*P* = 3.56 × 10^−7^, *n* = 28872) and 1.31 in the replication data (*P* = 0.45, *n* = 10256). In females, L2307F carriers had a lung cancer risk with ORs being 7.76 in the discovery data (*P* = 0.0002, *n* = 10919) and 3.22 in the replication data (*P* = 0.03, *n* = 4698). L2307F carriers had a significant 5.2-fold increased risk for lung adenocarcinoma (LAD) in the discovery data (*P* = 6.47 × 10^−9^, *n* = 19356) and a 2.5-fold increased risk in the replication data (*P* = 0.01, *n* = 7503). No associations of L2307F with the risk of lung squamous cell carcinoma (LSQ) (*n* = 16853) or small cell lung cancer (SCLC) (*n* = 14746) were observed in the discovery data. No L2307F variants were observed in LSQ or SCLC in the replication data. Colors indicate demographic and histological stratifications of the data. **b** Stratified analyses of the association between rs56009889 and LAD. Females who carried L2307F had a >8-fold greater risk of LAD in the discovery dataset (*P* = 0.0001, *n* = 8056) and a 4.7-fold risk of LAD in the replication data (*P* = 0.004, *n* = 3680). Never smoking females who harbored L2307F had a 7-fold greater risk of LAD in the discovery data (*P* = 0.01, *n* = 2817) and a 3.8-fold risk of LAD in the replication data (*P* = 0.15, *n* = 1212). **c** Distribution of L2307F homozygotes. All the homozygotes of L2307F in the discovery data, no matter what age, gender, and smoking status, developed LAD in the discovery data. No homozygotes were found in the replication data. **d** Higher ORs of association between rs56009889 and the risk of lung cancer, of LAD in overall and in females were found in Israeli (*n* = 1173) than in North Americas (*n* = 10858). All of the associations have reached significant. The upper 95% CI of the LAD risk in female in Israel (adjusted OR = 17.15; 95% CI 2.24–131.32, *n* = 373) was not shown because it was too high. Colors indicate stratifications of the data by histology and sex. The error bars are OR ± the 95% CI values. Source data are provided as a Source Data file.

rs56009889 maps within the *ATM* gene (Fig. 1a). This mutation results in L2307F missense mutation in the FAT domain that regulates ATM activity^13^, implying a putative functional role. Consistent with the location of this mutation in a highly conserved region that is critical for ATM function, in silico tools such as SNPeffect 4.0^14^ PolyPhen-2^15^ Fathmm-XF (http://fathmm.biocompute.org.uk/) and SIFT suggest a functional effect. Compared to non-carriers (C/C), L2307F carriers (T/C + T/T) had statistically significant increased risk of lung cancer in the discovery dataset (adjusted odds ratios (ORs = 4.19, *P* = 3.56 × 10^−7^), though the increased risk did not reach significance in the replication dataset (Table 2). Among females, L2307F was significantly associated with lung cancer risk with ORs being 7.76 (*P* = 0.0002) in the discovery dataset and 3.22 (*P* = 0.03) in the replication dataset. Among males, L2307F showed a weakly significant association with lung cancer risk in the discovery dataset and no association in the replication dataset (Fig. 2a and Supplementary Table 5). Stratification analysis by histology indicated that L2307F carriers had a significant 5.23-fold increased risk for lung adenocarcinoma (LAD) in the discovery dataset (*P* = 6.47 × 10^−9^) and a 2.48-fold increased risk in the replication dataset (*P* = 0.01), and there was little evidence for association with the risk of lung squamous cell carcinoma (LSQ) or of small cell lung cancer (SCLC) in either dataset (Supplementary Table 5). Females carrying L2307F showed an 8.05-fold (*P* = 0.0001) and 4.69-fold (*P* = 0.004) greater risk of LAD in the discovery and replication datasets, respectively (Fig. 2b and Supplementary Table 6). Notably, all the L2307F homozygotes (*N* = 5), no matter what age, gender, and smoking status, had LAD in this study (*P* = 0.004) (Fig. 2c). Moreover, the association exhibited a dose-response relationship between the number of mutated alleles and the LAD risk in the discovery dataset (*P*_trend_ = 5.44 × 10^−9^). A more significant role for L2307F in LAD pathogenesis than in LSQ or SCLC is reflected in the low mutant allele frequency of LSQ and SCLC in both datasets. These results suggested the association between rs56009889 and lung cancer risk was restricted to LAD and is especially prominent in women.

**Table 2:** Lung cancer risk for the carriers of *ATM*-L2307F (rs56009889) and *KIAA0930*-Q4X (rs150665432).

Outcome	Population	Gene	Genotype	Discovery Dataset				Replication Dataset				Meta-analysis^#^	
				No.		Adjusted^a^		No.		Adjusted^a^			
				Control	Case	OR (95% CI)	*P*	Control	Case	OR (95% CI)	*P*	OR (95% CI)	*P*
lung cancer	All	*ATM*	CC	13005	15767	1		5331	4891	1			
			TC	18	77	3.79 (2.2–6.6)	2.57E−06	15	19	1.31 (0.65–2.65)	0.45	2.52 (1.63–3.91)	3.18E−05
			TT	0	5	Inf (0.8–Inf)	0.068*	0	0	–	–	–	–
			TC + TT	18	82	4.19 (2.4–7.3)	3.56E−07	15	19	1.31 (0.65–2.65)	0.45	2.7 (1.75–4.16)	7.82E−06
			Trend				2.45E−07						–
lung cancer	Female	*ATM*	CC	5096	5777	1		2475	2203	1			
			TC	4	41	7.67 (2.6–22)	0.0002	5	15	3.22 (1.12–9.21)	0.03	4.94 (2.34–10.5)	2.92E−05
			TT	0	1	Inf (0–Inf)	0.49*	0	0	–	–	–	–
			TC + TT	4	42	7.76 (2.7–22)	0.0002	5	15	3.22 (1.12–9.21)	0.03	4.97 (2.35–10.5)	2.67E−05
			Trend				0.0002						–
LAD	All	*ATM*	CC	13005	6267	1		5331	2139	1			
			TC	18	61	4.68 (2.7–8.2)	7.92E−08	15	18	2.48 (1.22–5.04)	0.01	3.66 (2.36–5.69)	7.93E−09
			TT	0	5	Inf (1.9–Inf)	0.004*	0	0	–	–	–	–
			TC + TT	18	66	5.23 (3–9.2)	6.47E−09	15	18	2.48 (1.22–5.04)	0.01	3.93 (2.53–6.1)	9.96E−10
			Trend				5.44E−09						–
LAD	Female	*ATM*	CC	5096	2923	1		2475	1186	1			
			TC	4	32	7.91 (2.7–23)	0.0002	5	14	4.69 (1.65–13.4)	0	6.05 (2.86–12.79)	2.48E−06
			TT	0	1	Inf (0–Inf)	0.36*	0	0	–	–	–	–
			TC + TT	4	33	8.05 (2.8–23)	0.0001	5	14	4.69 (1.65–13.4)	0	6.1 (2.89–12.9)	2.14E−06
			Trend				0.0001						–
lung cancer	All	*KIAA0930*	GG	12642	14814	1		5308	4861	1			
			AG	126	355	2.41 (2–3)	7.83E−16	32	47	1.69 (1.05 -2.7)	0.03	2.27 (1.87–2.75)	1.9E-16
			AA	0	29	Inf (6.3—Inf)	2.29E−08*	0	0	–	–	–	–
			AG + AA	126	384	2.59 (2.1–3.2)	1.15E−18	32	47	1.69 (1.05 -2.7)	0.03	2.41 (1.99–2.92)	3.9E-19
			Trend				1.51E−19						–

^a^Adjusted for age at diagnosis/interview, gender, smoking status and PCs. ^#^Fixed-effects meta-analysis adjusted for age at diagnosis/interview, gender, smoking status and PCs.

*Values were generated from two-sided Fisher’s Exact Test. OR, 95% CI and *P* values generated from logistic regression model.

### Ethno-geographic lung cancer risk of *ATM*-L2307F

The *ATM*-L2307F was found in 4.43% (MAF = 0.023) individuals from lung cancer case control study from Israel, slightly higher in North Americans (MAF = 0.002) and close to monomorphic in other European countries (Supplementary Table 7). Consistent with this observation, rs56009889 is much more prevalent in the Ashkenazi Jewish population (https://gnomad.broadinstitute.org/variant/11-108196896-C-T). We therefore investigated if the association of rs56009889 and lung cancer risk was affected by country of origin. In both Israeli and North Americans, rs56009889 was significantly associated with the risk of lung cancer, of LAD in general and especially in women. However, the association was stronger in the Israeli case-control study than in North Americans (Fig. 2d) and the lack of variant carriers meant this analysis was not informative in Europeans. As shown in Supplementary Table 7, the ORs for LAD risk among L2307F carriers were 3.36 in North Americans (*P* = 0.004) and 6.74 in the Israeli case control study (*P* = 3.38 × 10^−6^). The female carriage of L2307F conferred an increased LAD risk with an OR of 3.81 in North Americans (*P* = 0.04) and 17.15 for the Israeli (*P* = 0.006). The replication data did not include a lung cancer study from Israel.

Because populations in Israel mainly include Jews and Arabs^16^, we then investigated whether L2307F had different prevalence and associations between the two ethnic groups as derived from genetic information (Supplementary Table 8). We observed that the L2307 occurred in 7.99% in Ashkenazi Jews from Israel (MAF = 0.042) and 8.53% in Ashkenazi Jews living in other countries (MAF = 0.045), but had very a low frequency in Arabian populations (Supplementary Table 9). In addition, although L2307F had a significant association with the risk of lung cancer and of LAD for Jews wherever they lived, the association was more marked in the Israeli Jews than Jews in other countries (Supplementary Table 8). Among L2307F carriers, the ORs for LAD risk were 7.86 in Israeli Jews (*P* = 2.12 × 10^−6^) and 3.40 for the Jews living in other countries (*P* = 0.005). Female Jews carrying L2307F had a 16.01-fold LAD risk (*P* = 0.008) in Israel and 4.23-fold risk in other countries (*P* = 0.03).

### *KIAA0930*-Q4X is suggestively associated with lung cancer

**Fig. 3:**
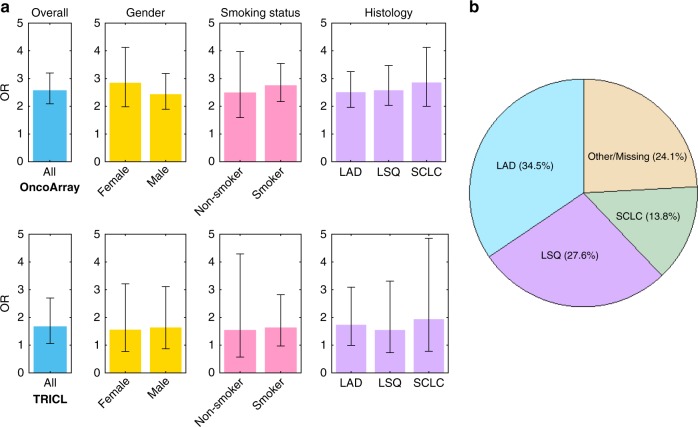
*KIAA0930* rs150665432 association with lung cancer risk. **a** Stratified analyses of the association between *KIAA0930* Q4X and lung cancer risk, shown by different colors. Compared to non-carriers, Q4X carriers had a significantly increased lung cancer risk with ORs being 2.59 in the discovery (*P* = 1.15 × 10^−18^, *n* = 27966) and 1.69 in the replication datasets (*P* = 0.03, *n* = 10248). Stratified analysis showed that Q4X carriers had an increased, consistent risk for lung cancer among females, males, smokers and non-smokers and consistent in histological subtypes. The error bars are OR ± the 95% CI values. *P* values were determined by logistic regression analysis adjusted by age, gender, smoking status and the principal components. Source data are provided as a Source Data file. **b** Distribution of *KIAA0930* Q4X homozygotes. In the discovery data, all homozygotes of the mutated allele in rs150665432 were developed to lung cancer in the discovery data. Source data are provided as a Source Data file (Source Data 1, 2, and 3). Color shades indicate the histological subtypes.

rs150665432, mapping within *KIAA0930* (Fig. 1b), is located at 22q13.31. The rs150665432 mutation codes for Q4X which results in the truncation of the full-length protein from 409 to 3 amino acids (https://www.ncbi.nlm.nih.gov/protein/NP_056079.1?report=graph). Compared to non-carriers (G/G), Q4X carriers (A/G + A/A) had an increased lung cancer risk in both the discovery (adjusted OR = 2.59; *P* = 1.15 × 10^−18^) and the replication datasets (adjusted OR = 1.69; *P* = 0.03) (Fig. 3a and Supplementary Table 10). Additionally, all Q4X homozygotes in the discovery set (*N* = 29) developed lung cancer (*P* = 2.29 × 10^−8^) (Fig. 3b), and the number of mutated alleles showed a dose-response relationship with lung cancer risk (*P*_trend_ = 1.51 × 10^−19^) in the discovery dataset (Table 2). No homozygotes were found in the replication dataset. Stratification analysis showed that Q4X had a significant risk in all strata: among females, males, smokers, non-smokers (Supplementary Table 10), and of LAD, LSQ, and SCLC (Supplementary Table 11) in the discovery dataset. In the replication, none of the strata reached significance, likely reflecting the small number of individuals with this uncommon variant in the subset analyses. Although the frequency of rs150665432 in controls varies non-significantly among geographic populations, ORs of the association between Q4X and lung cancer risk were higher in North American Countries (adjusted OR = 4.19; *P* = 3.27 × 10^−16^) than in European Countries (adjusted OR = 1.65; *P* = 0.0003, Supplementary Table 12).

### Mutations and onset age of lung cancer

**Fig. 4:**
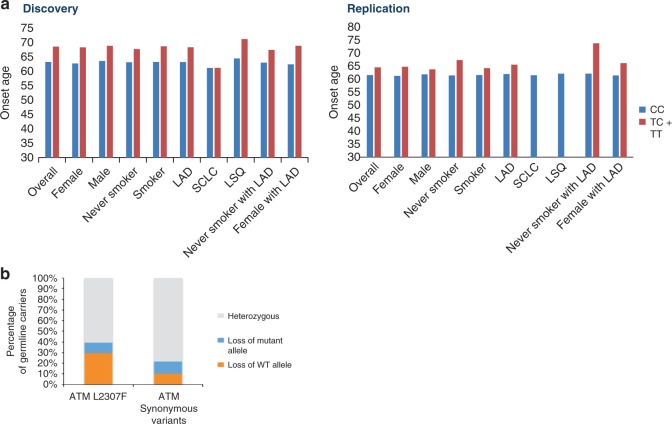
The onset of lung cancer risk and biallelic two-hit events of *ATM* rs56009889. **a** rs56009889 affects the age of onset. The error bars are mean + the standard error of the mean (SEM). In the discovery data, the mean age of onset for lung cancer cases carrying L2307F was significantly higher than cases of non-carriers. Later age of onset was observed for overall lung cancer (*n* = 15830), females (*n* = 5810), males (*n* = 10019), smokers (*n* = 14006), LAD (*n* = 6329) and females (*n* = 2954) with LAD. In the replication data, a borderline significant difference in the age of onset was observed only in females with LAD (*n* = 906) and non-smoker with LAD (*n* = 293) though the sample size is small. *P* values were determined by the two sides *t* test without adjustment. No carrier of the T allele developed LSQ and SCLC in the replication data. **b** the rate of loss of heterozygosity targeting either *ATM* L2306F allele or synonymous variants in *ATM* gene. Source data are provided as a Source Data file.

Carriers of L2307F tended to be significantly older at lung cancer onset overall and in all subsets except small-cell and squamous lung cancer in the discovery data. For female lung AD, this effect was most pronounced in the discovery dataset (69.37 ± 10.71 vs 62.78 ± 11.06, *P* = 0.0007) and showed borderline significance in the replication dataset (68.74 ± 10.49 vs 63.69 ± 10.31, *P* = 0.09, Supplementary Table 13 and Fig. 4a). As *ATM* is more highly expressed at older age^17^ the variant effects may be more pronounced at older ages, but larger datasets are needed to validate this observation. No consistent variation in age by genotypes of rs150665432 was found overall or in subgroups (Supplementary Table 14).

### Biallelic two-hit events of *ATM*-L2307F

To understand the interplay of *ATM*-L2307F and the somatic mutation profiles, we utilized the germline and matched tumor data for 2127 lung cancer patients of European ancestry in an additional, independent MSK-IMPACT dataset (Supplementary Table 15). In this study, germline L2307F was observed in 63 (3%) cases. The frequency of germline L2307F was higher in LADs (*P* = 0.0009), females vs male patients (*P* = 0.03), light-smokers (≤5 pack years) vs heavy smokers (>5 pack years, *P* = 0.003) and patients with EGFR oncogenic mutations vs cases without EGFR oncogenic mutations (*P* = 0.001). 61 of 63 patients were L2307F heterozygotes, and of these 29.5% patients showed loss of heterozygosity (LOH) of the wild-type allele compared to 10% background LOH (*P* = 0.0026) (Fig. 4b). *KIAA0930* was not a gene included in the 468 cancer- associated genes targeted by the MSK-IMPACT study and were unable to explore germline somatic interactions for this gene in this dataset.

### Mutation-related isoforms expression in lung tissue

**Fig. 5:**
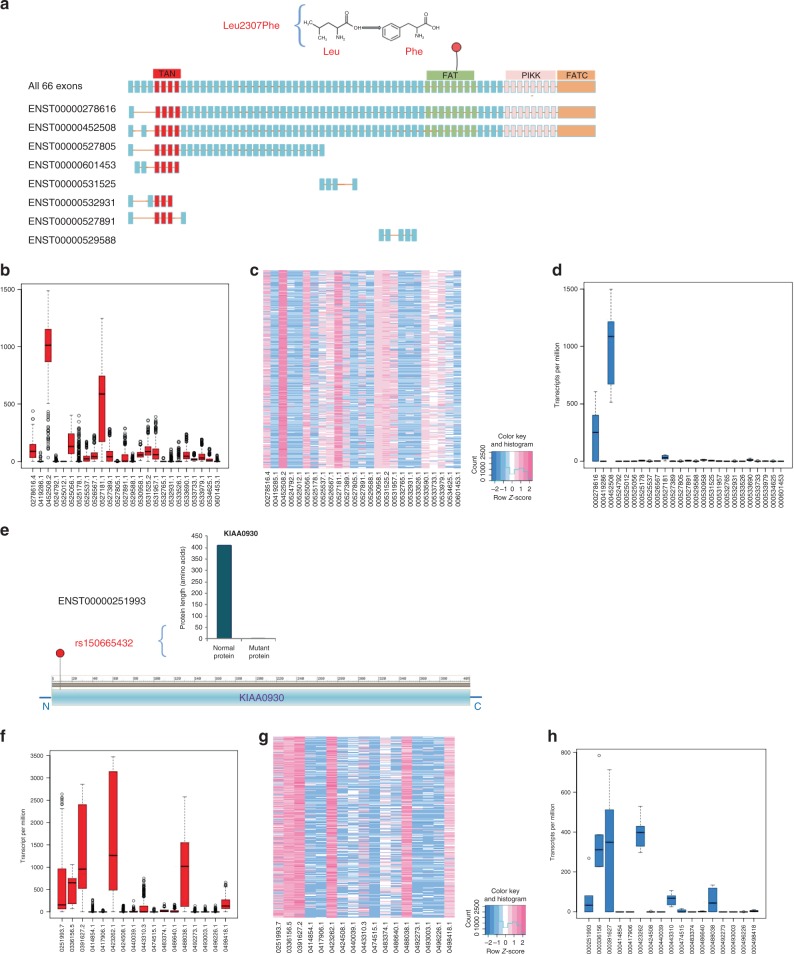
ATM and KIAA0930 isoforms. **a** ATM has eight isoforms that produce proteins with different length. ATM has four functional domains, including TAN domain which is used for telomere-length maintenance and DNA damage repair, FAT and FATC domains, which are important regulatory domains, and Phosphoinositide 3-kinase related kinase (PIKK), which is a catalytic domain that has intrinsic serine/threonine kinase activity. **b** *ATM* isoforms expression from GTEx data (*n* = 427). **c** A heatmap showing *ATM* isoforms expression from GTEx data. **b** *ATM* isoforms expression from Germany data (*n* = 6). **e** ENST00000251993 is the full-length and canonical isoform of KIAA0930. rs150665432 can truncate its protein length from 409 to 3 aa. **f** *KIAA0930* isoforms expression from GTEx data (*n* = 427). **g** A heatmap showing *KIAA0930* isoforms expression from GTEx data. **h** *KIAA0930* isoforms expression from Germany data (*n* = 6). Boxplots in this figure were the visualization representing three quartiles (25%, median: 50%, and 75%) of the data set that are calculated using the percentile function, and the minimum and maximum values of the data set that are not outliers. Outliers are detected using the interquartile range method. Data points are labeled as outliers if they lie 1.5 times the interquartile range above or below the end points of that range.

Both *ATM* and *KIAA0930* have alternative splicing in various tissues, which might cause certain exons of a gene to be excluded and thus fail to translate to amino acid sequence in the related isoforms. To clarify whether rs56009889 and rs150665432 are included in the isoforms that are normally expressed in lung tissue and thereby may play a functional role in lung, we investigated the isoform expression of *ATM* and *KIAA0930*. ATM has eight isoforms encoding produce proteins with different length. L2307F causes an amino acid (aa) change in the two isoforms ENST00000278616 and ENST00000452508, producing the full-length isoforms of ATM protein comprising 3056 aa long that include TAN, FAT, FATC, and Phosphoinositide 3-kinase related kinase (PIKK) domains (Fig. 5a). The Genotype-Tissue Expression (GTEx) project (Supplementary Table 16 and Fig. 5b–c) and expression data from Germany (Supplementary Table 17 and Fig. 5d) implied that that both ENST00000278616 and ENST00000452508 are expressed in normal lung tissue.

KIAA0930 has 10 isoforms that can produce proteins of varying lengths, among which ENST00000251993 is the full-length and canonical isoform (https://gnomad.broadinstitute.org/gene/ENSG00000100364/transcript/ENST00000251993). rs150665432 truncates the protein length of ENST00000251993 from 409 to 3 amino acids (Fig. 5e) and shortens the protein length of another abundant isoform, ENST00000492273, from 85 to 3 amino acids. In addition, rs150665432 is included in 4 other isoforms, including ENST00000417906, ENST00000488038, ENST00000486640 and ENST0000049622, which are untranslated transcripts. The ENST00000251993 and ENST00000488038 transcripts, which include rs150665432, are the primary transcripts expressed in lung tissue in GTEx data (Supplementary Table 18 and Fig. 5f–g), as well as in the data from Germany (Supplementary Table 19 and Fig. 5h).

### Gene expression in lung cancer and multiple cancer types

We explored the role of the uncharacterized protein (http://www.uniprot.org/uniprot/Q6ICG6) *KIAA0930* in lung cancer pathogenesis by investigating whether its expression was associated with lung cancer development, comparing its expression to *ATM*, a tumor suppressor protein. *KIAA0930* was significantly over-expressed in LAD (*P* = 0.004) and LSQ (*P* = 1.62 × 10^−12^) in The Cancer Genome Atlas (TCGA) (Supplementary Fig. 2A–D). *KIAA0930* showed significant over-expression in lung cancer compared to normal lung samples in an independent dataset from Harvard^18^ (*P* = 0.0005), while *ATM* showed limited variability (Supplementary Fig. 3 and Supplementary Table 20). Additionally, we observed *KIAA0930* expression was significantly upregulated in the majority of carcinomas developing from epithelial cells (Supplementary Table 21), suggesting that *KIAA0930* is a carcinoma-associated candidate gene.

## Discussion

Carcinogenesis is a stepwise process characterized by the accumulation of mutations, including germline and somatic alternations^19^. Identification of cancer-related driver germline mutations can provide targets for personalized cancer screening, prevention^9,10^ and treatment and drug development^20^. A challenge in analysis of rare variants is having enough samples to accurately identify genotypes linked with genetic susceptibility. Here, we have used a large-genotyping resource in a two-phased study; a discovery phase to identify potential variants, followed by a replication phase to confirm the fidelity of genotyping and to evaluate the robustness of observed differences between cases and controls. Using this approach, we report large-effect associations with two variants; a rs56009889 germline mutation, where we observed a reproducible association in LAD and a suggestive association with the rs150665432. A case only analysis in the MSK-Impact study of rs56009889 reinforced the link between this polymorphism and LAD and particularly in females.

Both rs56009889 and rs150665432 are coding mutations, a missense variant in *ATM*-L2307F and a stop-gain variant *KIAA0930*-Q4X and both appear included in the full-length isoforms of genes. Individuals homozygous for *ATM* germline mutations can develop Ataxia Telangiectasia, which includes susceptibility to cancer within its disease spectrum, and mutations in *ATM* in a heterozygote state have been implicated in cancer susceptibility^21^. The functional impact of *ATM*-L2307F remains ambiguous; in silico predictions suggest this variant may impact function, but it is reported by Clinvar as “likely benign” or “benign” (https://www.ncbi.nlm.nih.gov/clinvar/variation/127430/) and has not been associated with Ataxia Telangiectasia. Here, we observed a strong association between this variant and LAD and particularly women. *ATM*-L2307F was found to have high prevalence in Ashkenazi Jews and the association with lung cancer was stronger in a population from Israel. Similar to harmful *BRCA* founder mutations that also have high prevalence in Ashkenazi Jews^22^ and are used in clinical practice^23^ the association between *ATM*-L2307F and LAD may be clinically relevant to this population.

Mechanistically, L2307F is predicted to be deleterious by in silico analysis and defective ATM proteins are known permit the accumulation of new mutations^24^. *ATM* is a tumor suppressor gene, can recognize and repair damaged or broken DNA strands, and help maintains the stability of other genes (https://ghr.nlm.nih.gov/gene/ATM). It is noteworthy that all L2307F homozygotes had LAD and we observed an excess of LOH in tumors of allele carriers relative to non-carriers implying that biallelic loss might be important in this process. TCGA also reported that the most frequent pathogenic germline variants in LAD were heterozygous variants in *ATM*, occurring in aggregate among 1.2% of cases^25^. The Tumor Sequencing Project, investigating 188 LAD cases, also found that *ATM* was one of the most common genes that somatically mutated in LAD^26^. It remains to be tested if variants in other populations also contribute to lung cancer susceptibility, or if L2307F co-occurring with other clearly pathogenic *ATM* mutations increases lung cancer risk beyond that experienced by heterozygotes.

We additionally identified a suggestive association of *KIAA0930*-Q4X with increased risk for lung cancer. KIAA0930 is an uncharacterized protein (http://www.uniprot.org/uniprot/Q6ICG6) and its function has not been fully investigated. The rs150665432 - Q4X mutation appears to comprise a loss of function allele, which is included in the full-length isoform of KIAA0930. This gene is expressed in normal lung, and *KIAA0930* expression is significantly upregulated in lung cancer and other carcinomas developing from epithelial cells, suggesting *KIAA0930* might play a role in the development of those carcinomas. Also, data in The Human Protein Atlas showed that *KIAA0930* expression significantly affects survival in patients with carcinomas (https://www.proteinatlas.org/ENSG00000100364-KIAA0930/pathology), such as liver, renal or endometrial cancer, which also supports a role of *KIAA0930* as a carcinoma-associated candidate gene. Nevertheless, this association must be studied further to ensure its robustness and the mechanism by which the stop-gain mutation Q4X increases risk remains unclear.

In conclusion, we have used large-case control and case only collections of lung cancer to discover and validate high-risk, low-prevalence germline mutations. Elements of our study design, such as replicating results in an independent dataset, analyzing the data by geographic populations and ethnicities, confirming the genotyping fidelity, comparing MAFs of the mutations in our datasets to those in public sequencing datasets, in silico analysis and performing LOH exploration contribute to the robustness for our results. The elevated genetic risks associated with these variants imply potential clinical benefits in using these variants for the identification of individuals who would benefit most from screening programs, as well as suggestions for therapeutic targets. The identification of the novel lung cancer-related germline mutations could greatly advance our understanding of lung cancer etiology.

## Methods

### Study subjects

The OncoArray consortium, which was used in the discovery phase, is a network created to increase understanding of the genetic architecture of common cancers. The Dartmouth component of the Oncoarray consortium used genotyping data from 57,776 samples, obtained from 29 lung cancer studies across North America and Europe, as well as Asia^27^, along with additional samples from head and neck cancer patients that were included to improve genotype calling for rare variants. The OncoArray consortium participants who were lacking disease status (because they were not part of the lung cancer-related studies), who were close relatives (second-degree relatives or closer) or who were duplicate individuals or other subjects, or who had a low call rate of genotype data, or who did not pass quality control (QC), or who were non-European, were excluded from the current study. There were 5742 participants in the OncoArray consortium who were also genotyped in the replication phase, and therefore these samples were excluded from the analysis in the discovery phase. Finally, 28,878 European-descent participants from 26 studies, including 15,851 lung cancer cases and 13,027 healthy controls, were included in the discovery dataset of the current case-control study.

The 25 studies in the current discovery dataset included the Alpha-Tocopherol Beta-Carotene Cancer Prevention Study (ATBC), Canadian screening study (CANADA), Cancer de Pulmon en Asturias (CAPUA), Copenhagen lung cancer study (COPENHAGEN), Environment and Genetics in Lung Cancer Study Etiology (EAGLE), The Carotene and Retinol Efficacy Trial (FHCRC), Liverpool Lung Cancer Project (FIELD), German lung cancer study (GERMANY), Harvard Lung Cancer Study (HSPH), The IARC L2 Study (IARC), Israel study (ISRAEL), The Kentucky Lung Cancer Research (KENTUCKY), MD Anderson Cancer Center Study (MDACC), The Malmö Diet and Cancer Study (MDCS), Multiethnic Cohort Study (MEC), New England Lung Cancer Study (NELCS), The Nijmegen Lung Cancer Study (NIJMEGEN), Norway Lung Cancer Study (NORWAY), Northern Sweden Health and Disease Study (NSHDC), The Prostate, Lung, Colorectal and Ovarian Cancer Screening Trial (PLCO), RESOLUCENT study (RESOLUCENT), Tampa Lung Cancer Study (TAMPA), Total Lung Cancer: Molecular Epidemiology of Lung Cancer Survival (TLC), The Mount-Sinai Hospital-Princess Margaret Study (TORONTO), The Vanderbilt Lung Cancer Study (VANDERBILT), whose details were shown in Supplementary Table 22. Among the 26 studies, 13 studies, including ATBC, CAPUA, COPENHAGEN, EAGLE, FIELD, IARC, MDCS, NIJMEGEN, NORWAY, ISRAEL, NSHDC, GERMANY and RESOLUCENT, obtained samples from Europe. Another 13 studies, including CANADA, FHCRC, HSPH, KENTUCKY, MDACC, MEC, NELCS, PLCO, TAMPA, TLC, TORONTO, and VANDERBILT, recruited subjects from North America.

We used the Affymetrix Axiome array study^11^ from the Transdisciplinary Research in Cancer of the Lung consortium in the replication phase. The Affymetrix Axiome array study was a large-pooled sample, assembled from 10 independent case-control studies, including Mount-Sinai Hospital-Princess Margaret (MSH-PMH), Multiethnic Cohort, Liverpool Lung Project, Nurses’ Health Study and National Physicians Health Study, the European Prospective Investigation into Cancer and Nutrition (EPIC) Lung, the Prostate, Lung and Ovarian Cancer Screening Trial, Carotene and Retinol Efficacy Trial, Russian Multi-Cancer Case-Control Study, Melbourne Collaborative Cohort Study and Harvard Lung Cancer Study. Of the 12651 subjects in the Affymetrix Axiome array study, the participants who were lacking disease status, or who were non-European, or whose samples had lower call rate (missing genotype calls >0.05), were excluded. Finally, the replication dataset of the current case-control study comprised 10,268 European-descent participants, including 4916 lung cancer cases and 5352 healthy controls.

All studies were reviewed and approved by institutional ethics review committees at the involved institutions.

### Demographic characteristics

Descriptive statistical analyses were conducted to characterize the study population of lung cancer cases and controls in both discovery and replication datasets. The difference between cases and controls in the distribution of age at diagnosis, gender and smoking status were evaluated using the *χ*^2^ test. Statistical analyses were performed with Statistical Analysis System software (Version 9.3). Principal component analysis (PCA) was performed based on GWAS data with the EIGENSTRAT program for both discovery and replication datasets (Supplementary Fig. 4A–B), respectively. To calculate these principal components (PCs), we analyzed GWAS data after excluding the sex chromosomes, variants with MAF less than 0.05 and after sampling SNPs that were uncorrelated with each other.

### Association analysis

We performed association analyses for the mutations having Minor Allele Frequencies (MAF) <0.01. Case-control association tests for genotyped data were conducted using 1-degree-of-freedom Cochran-Mantel-Haenszel tests with the application of PLINK version 1.9 to discover the germline mutations with large effects on lung cancer risk. In order to investigate the mutations that altered protein sequence, we only keep the mutations within the exome to do further analysis.

To infer Jewish versus Arabic Ancestry in the study from Israel, we used the program AIPS^28^ (https://morgan1.dartmouth.edu/~f000q4v/html/aips.html), which enables us to infer ancestry membership using a distance-based analysis to account for geogenetic subpopulation structure. The analysis includes populations of known origin from 22 European populations including Ashkenazi Jewish, and Palestinian, Druze and Bedouin populations who were labeled as Arab. The detailed results were shown in Supplementary Table 9.

We estimated the association between the risk of lung cancer and the selected germline mutations by computing the ORs and 95% confidence intervals (CIs) in univariate and multivariate logistic regression analyses in both datasets. In the multivariate logistic regression model, OR and 95% CI were adjusted by age, gender, smoking status (never and ever) and the PCs. To control for possible population structure, we adjusted for three PCs in the discovery dataset because the *P* values of anova statistics for population differences between Control and Case were 2.18E-05, 0.0001 and 0.018 for 1st PC, 2nd PC and 3rd PC respectively. We adjusted for two PCs in the replication data set for which there were no eigenvectors that varied significantly between cases and controls (The *P* value of anova statistics for population differences between Control and Case for 1st PC and 2nd PC were 0.287 and 0.189, respectively.).

We further stratified the association of the selected germline mutations and lung cancer risk by gender and smoking status. We also estimated the association between the selected SNP variants and the risk of LAD, lung squamous cell carcinoma or small cell lung cancer, respectively, in univariate and multivariate logistic regression analyses. A full listing of variants that we identified in the discovery phase by histology is provided in supplementary Table 23. There were four mutations including rs17843743, 3:9970073, rs150665432, and rs61816761 with small cell lung cancer that reached the criteria of *P* values of less than 5.0 × 10^−8^ and OR values of more than 2.0 in the discovery dataset. However, among unaffected individuals, the MAFs of none of the new mutations in replication dataset were comparable to those in discovery dataset.

We further stratified the association in the discovery dataset by geographic populations in univariate and multivariate logistic regression analyses. Based on the MAF of rs56009889 and the location of the study sites in the discovery dataset, we categorized all the studies to three subgroups, including Israeli among which rs56009889 had the highest MAF, population in other European countries, and North Americans. We then investigated the association in Jews of the discovery dataset by geographic populations in univariate and multivariate logistic regression analyses. Since the frequency of rs150665432 in controls varies non-significantly between geographic populations, we categorized all the studies to two subgroups, including population in European countries and North American countries, to calculate the associations of rs150665432 and lung cancer risk in different geographic populations. Statistical analyses for were performed with SAS 9.3 in both discovery and replication phase; a *p*-value of <0.05 was considered to be significant.

Meta-analyses were performed with the application of R package ‘meta’ (http://www.imbi.uni-freiburg.de/lehre/lehrbuecher/meta-analysis-with-r) that combined test statistics and standard errors across studies. A fixed effect model was used to combine the studies in meta-analysis.

### Genotyping, quality control, and the reliability

A novel technology, developed by Illumina to facilitate efficient genotyping was used to genotype OncoArray samples^27^. Quality control steps follow the approach described previously for the OncoArray^29^. Briefly, samples with low-genotyping rates and poor genotyping assays (judged by success rate, or genotype distributions that deviated from expectation by Hardy Weinberg equilibrium) were excluded based on Standard quality control. SNPs, showing departure from Hardy-Weinberg equilibrium in the controls (*P*-value < 1 × 10^−6^) or lower call rate (missing genotype calls >0.05) or samples with less than 95% call rate were excluded. 533,631 variants for OncoArray samples passed quality control procedures and were included as valid markers, of which 105,736 variants, whose MAF was <0.01 were rare variants. Genotyping 395,745 SNPs from samples of the Affymetrix study was performed using a custom Affymetrix Axiom Array (Affymetrix, Santa Clara, CA, USA), which contains a comprehensive panel of key GWAS markers, rare and low-frequency variants and indels^11^. The datasets were built using the Genome Reference Consortium Human build 37.

In order to validate the reliability of genotyping data, we compared the MAFs of the selected germline mutations in unaffected individuals of the discovery and the replication datasets, respectively, to those in public sequencing projects or datasets including the Exome Aggregation Consortium (ExAC)^12^, the NHLBI GO Exome Sequencing Project (GO-ESP) and the Trans-Omics for Precision Medicine (TOPMed) Program. ExAC is a released public exome sequencing dataset with variations on 60,706 unrelated individuals. GO-ESP is an exome sequencing project that included European American and African American participants. TOPMed sequenced the DNA of people from diverse ethnic backgrounds, with 50% being of European descent and 30% of African descent.

### Concordance analysis

We confirmed the genotyping fidelity of the selected germline mutations in the OncoArray platform and the Affymetrix platform, respectively, by considering the concordance of these genotypes between the two platforms. A total of 5742 subjects in the OncoArray consortium were duplicate individuals in the Affymetrix data. Even though the 5742 subjects were excluded in the discovery dataset and included in the replication dataset, we calculated the concordance of genotyping between the OncoArray consortium and the Affymetrix study for the selected germline variants in the 5742 individuals whose genotyping results were available for both platforms.

The concordance rate was based on the agreement between OncoArray genotyping and Affymetrix genotyping, and we considered the general concordance and concordance between the rare alleles only^29^. Supplementary Table 24 describes the genotype frequencies in different situations of agreement between OncoArray genotyping and Affymetrix genotyping.

The general concordance rate was estimated using the genotype frequencies, which were in agreement between OncoArray genotyping and Affymetrix genotyping, incorporating all genotype frequencies (*n*).

General concordance = (*a* + *e* + *i*)/*n*.

The concordance of rare alleles was estimated using the genotype frequencies of the minor/minor and major/minor, which were in agreement between Oncoarray genotyping and Affymetrix genotyping, incorporating all genotype frequencies other than the genotype frequency of major/major. Concordance of rare Allele = (*i* + *e*)/(*n*−*a*).

### Analysis of the differences in age

Student *t*-test was used to evaluate the differences in age at onset of lung cancer between different genotypes of the selected germline mutations in cases. We then evaluated the differences in age at onset with stratified by gender, smoking status and histology of lung cancer.

### Germline-somatic integrated analysis

A dataset was comprised of 2686 advanced lung cancer patients who were recruited at Memorial Sloan Kettering Cancer from January 2014 until May 2016. In this dataset, 2127 cases were of European ancestry and selected for the current analysis. The frequency of Jewish heritage in this population is reported to be about 18%, but we did not have sufficient information from the targeted sequencing panel to infer ancestry. The germline analysis was performed in an anonymized method using a deterministic hash algorithm and samples were assigned a unique identifier to link germline and somatic data for integrated analysis. Tumor and blood DNA from patients were sequenced by Integrated Mutation Profiling of Actionable Cancer Targets (MSK-IMPACT). This assay captures the coding exons and select introns of 468 cancer- associated genes. LOH in the tumor was evaluated for total, allele-specific, and tumor purity and ploidy, using FACETS version 0.5.6. The background LOH rate was estimated using synonymous variants in the same gene. Statistical significance was computed using fisher exact test.

### RNA-seq and splicing analysis

We performed mRNA-seq analysis with using RNA-seq data from the human normal lung tissues that were adjacent of human lung invasive mucinous adenocarcinoma of six patients. The patients were enrolled from Germany, so that the data was called Germany data in this study. The RNA sequencing reads were obtained by high throughput sequencing and downloaded from Gene Expression Omnibus (GEO). We used bowtie2 to align RNA sequencing reads with quantifying isoform abundances with RSEM v1.2.22. In addition, to confirm the isoform expression of *ATM* and *KIAA0930* in lung, we also used lung tissue-specific Isoform expression values from the GTEx v7 dataset using RSEM. All of 427 lung samples that had isoform expression values are used. We plot raw isoform quantification values with R. After isoform-level transcripts per million (TPM) estimates were transformed via log2 (1 + TPM), hierarchical clustering was performed on the correlation matrix in R, using the heatmap.2 package from gplots version 3.0.1.1.

### Structure-based prediction

With using SNPeffect 4.0^14^, we explored TANGO that is a statistical mechanics algorithm to predict protein aggregation based on the physics-chemical principles of β-sheet formation^30^. PolyPhen-2^15^ was applied to predict the functional effects of the germline mutations. We used Fathmm-XF^18^ to perform accurate prediction of the functional consequences of the mutations with applying machine learning method.

### Gene expression

TCGA level 3 RNA-seq data and clinical patient data related to 19 cancer types, composing of LAD that included 515 tumor samples and 59 normal samples, lung squamous cell carcinoma (LUSC) that included 503 tumor samples and 52 normal samples, bladder urothelial carcinoma (BLCA), breast invasive carcinoma (BRCA), cervical squamous cell carcinoma (CESC), cholangiocarcinoma (CHOL), colon adenocarcinoma (COAD), esophageal carcinoma (ESCA), glioblastoma multiforme (GBM), head and neck squamous cell carcinoma (HNSC), kidney renal clear cell carcinoma (KIRC), kidney renal papillary cell carcinoma (KIRP), liver hepatocellular carcinoma (LIHC),pancreatic adenocarcinoma (PAAD), prostate adenocarcinoma (PRAD), rectal adenocarcinoma (READ), Sarcoma, Thymoma, thyroid carcinoma (THCA), were used to investigate whether or not the expression of *ATM* or *KIAA0930* were associated with the primary cancer. In all, 7570 samples, including 6930 tumor samples and 640 normal samples, were included in the analysis. The significance of difference in gene expression levels between tumor and normal samples was estimated by comparing generating Transcripts per million (TPM) expression values, employing UALCAN to perform *t*-test^31^.

Harvard lung expression data^18^ included the mRNA expression values for 12,600 genes that was rescaled and normalized from the raw expression data by a rank-invariant scaling method, in order to removing the batch differences. A total of 203 samples, including 127 LADs, 21 lung squamous cell carcinomas, 20 lung carcinoids, 6 small cell lung cancer and 17 normal lung specimens, were consisted in the study and performed with microarray analysis. Of the 12,600 genes, both *ATM* and *KIAA0930* were included. Additionally, exon 2 and 5 of *ATM* were analyzed without corresponding whole gene expression changes. We used *t*-test to evaluate the differences in gene expression levels of *ATM* exon 2 and 5 and *KIAA0930*, respectively, between lung cancer and normal lung samples, in general and within each histologic type.

### Reporting summary

Further information on research design is available in the Nature Research Reporting Summary linked to this article.

## Supplementary information

Supplementary Information

Reporting Summary

## Data Availability

The data that support the findings of this study are available. The access numbers are “phs001273” for Oncoarray study, “phs001681.v1.p1” for the Affymetrix study, and “phs001783.v1.p1” and “phs001858.v1.p1”for the MSK-IMPACT study in dbGAP. The source data underlying Figs. 1–4 are provided as a Source Data file.

## Source Data

Source Data

## Code availability

No unreported software and custom computer code were used to generate results.
