# Supplementary material for: Protein-altering germline mutations implicate novel genes related to lung cancer development
Source: Nat Commun. 2020 May 11;11:2220. doi: 10.1038/s41467-020-15905-6 (PMC7214407; doi:10.1038/s41467-020-15905-6)
Supplement: Supplementary file 1 — Supplementary Information [file 41467_2020_15905_MOESM1_ESM.pdf]

Protein-altering Germline Mutations Implicate Novel Genes Related to Lung Cancer Development

Ji et al.

1 Association Analysis in Discovery dataset.

- Discovery dataset comprised 28,878 individuals of European ancestry.
- To identify the mutations within the exome that had association $P < 5.0 \times 10^{-8}$ and OR > 2.0
- Using the basic association test without adjustment.
- rs56009889, rs150665432, and rs61816761 were found in this phase

2 Confirm the genotyping fidelity in both datasets

- Comparing the concordance of the selected mutations with using the 5742 individuals who were genotyped using both genotyping platforms
- Comparing MAFs of the selected mutations in both datasets with their MAFs in public datasets

3 Histologic and Gender-specific Lung Cancer Risk

- Identifying the risk of lung cancer for the carrier of the selected mutations
- Stratification analysis by histology and gender
- Discovery dataset comprised 28,878 individuals and replication dataset comprised of 10,268 European ancestry.

4 Ethno-geographic distribution and Lung Cancer Risk

- To investigate whether or not the selected mutations had different ethno-geographic distribution
- To indentify the possible different lung cancer risk during various populations.

5 Mutations and Onset Age of Lung Cancer

- To investigate whether or not the selected mutations were associated with the onset age of lung cancer

6 Biallelic two-hit events of ATM-L2307F

- To understand the interplay of *ATM*-L2307F and the somatic mutation profiles
- Using the germline and matched tumor data for 2,127 lung cancer patients of European ancestry in an additional, independent MSK-IMPACT dataset

7 Mutation related Isoforms Expression in Lung Tissue

- To clarify whether the selected mutations are included in the isoforms that are normally expressed in lung tissue and thereby may play a functional role in lung,
- Using GTEx dataset with 427 lung samples and Germany dataset with 6 samples

8 Gene Expression Analysis

- Investigating whether gene expression was associated with lung cancer risk and other cancer risk
- Using Harvard lung expression data with 203 samples and TCGA level 3 RNA-seq data with A total of 7570 samples, including 6930 tumor samples and 640 normal samples

Supplementary Figure 1. Schematic overview of the study design.

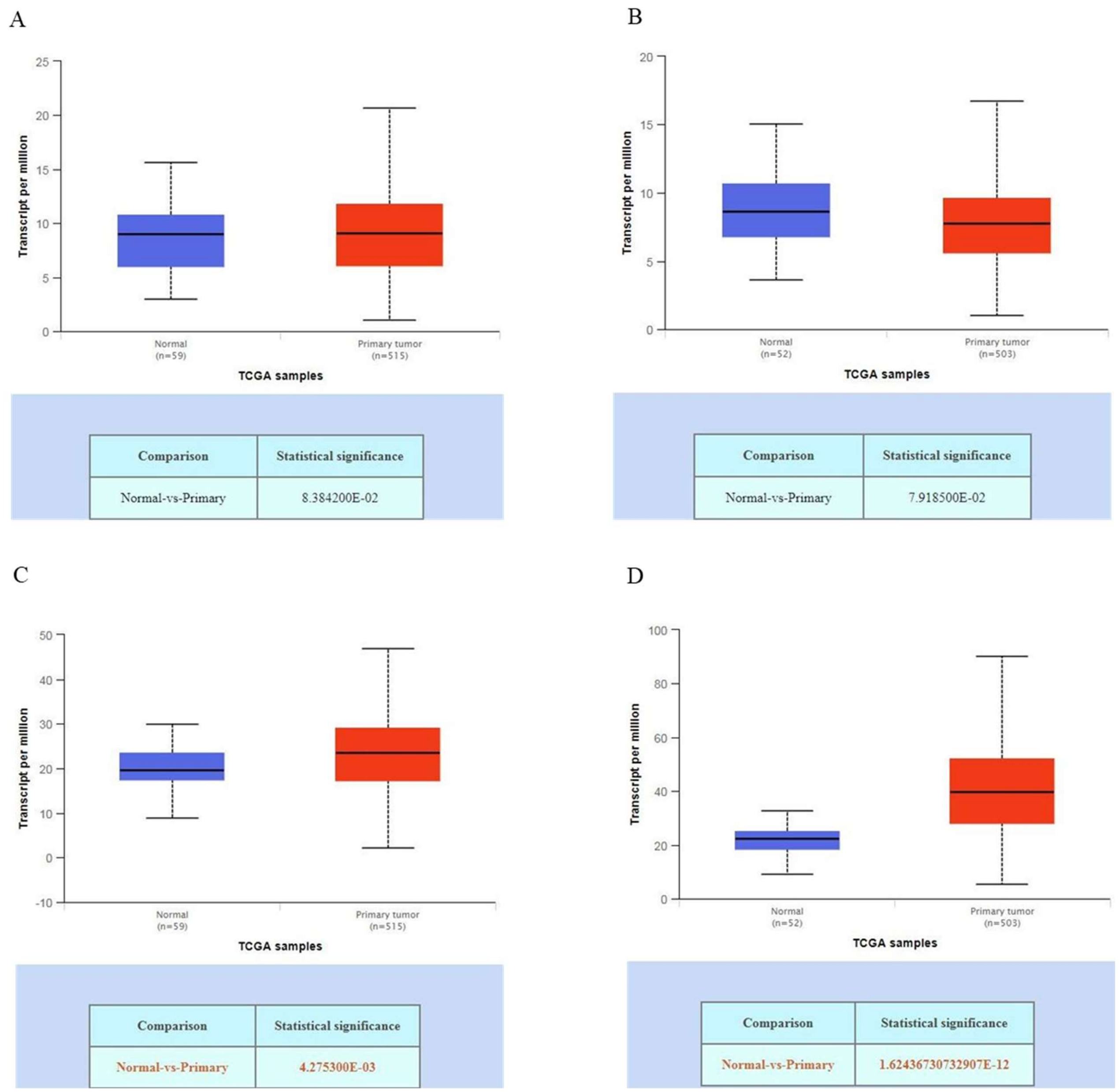

Supplementary Figure 2. *ATM* and *KIAA0930* gene expression in TCGA**.** The visualizations represent interquartile range (IQR) including minimum, 25th percentile, median, 75th percentile and maximum values. *P* values were determined by the two sides t test without adjustment. **A,** *ATM* expression for LAD in TCGA. **B,** *ATM* expression for LSQ in TCGA. **C,** *KIAA0930* expression for LAD in TCGA. *KIAA0930* is significantly over-expressed in LAD than in normal lung tissue. **D,** *KIAA0930* expression for LSQ in TCGA. *KIAA0930* is significantly over-expressed in LSQ than in normal lung tissue.

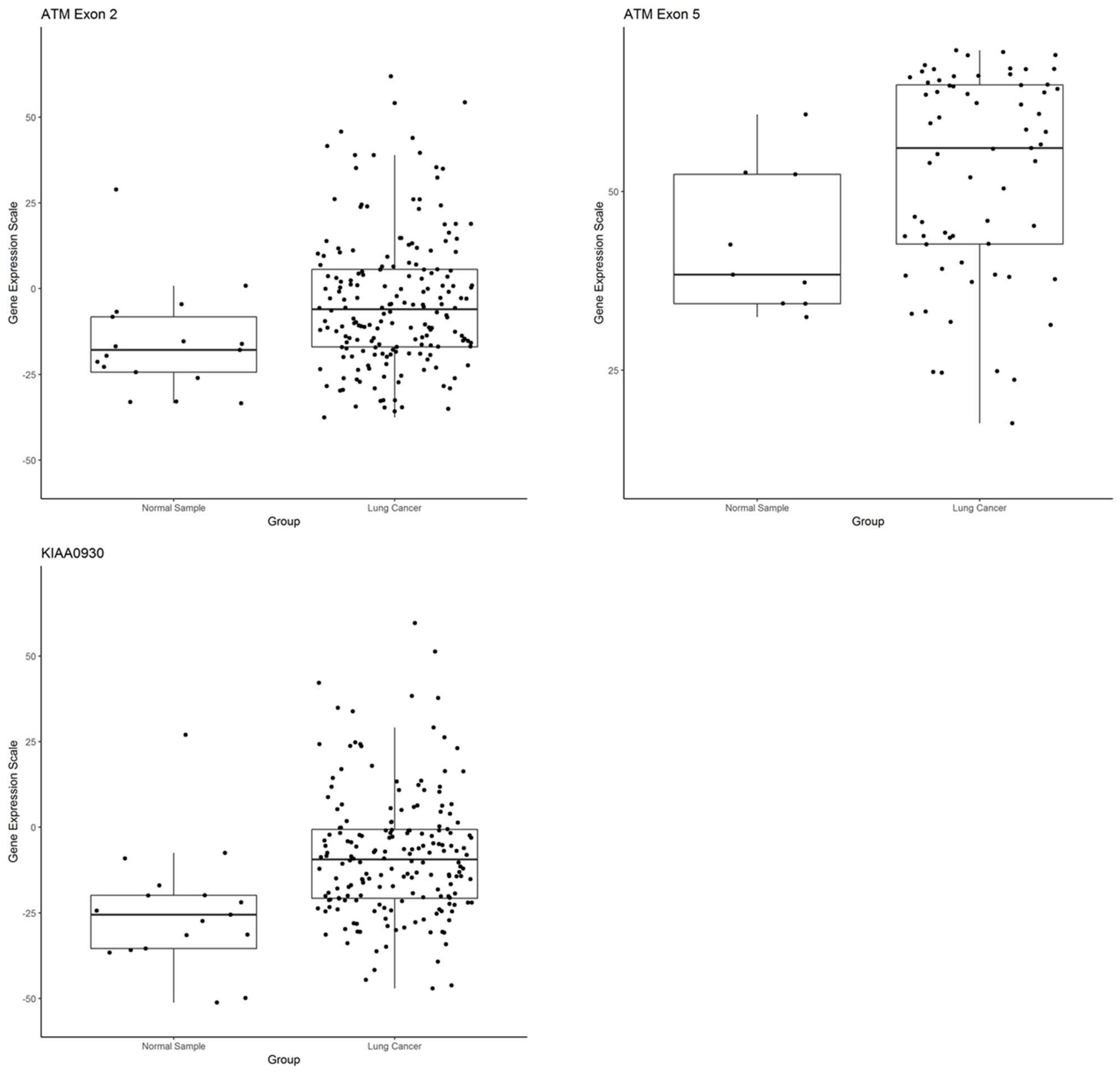

Supplementary Figure 3. *ATM* and *KIAA0930* gene expression for lung cancer in Harvard Data. The expression of *ATM* Exon 2 whose ID is ENSE00001467894, *ATM* Exon 5 whose ID is ENSE00002194724 and *KIAA0930* were shown. Boxplots in this figure were the visualization representing three quartiles (25%, median: 50%, and 75%) of the data set that are calculated using the percentile function, and the minimum and maximum values of the data set that are not outliers. Outliers are detected using the interquartile range method. *P* values were determined by the two sides t test without adjustment.

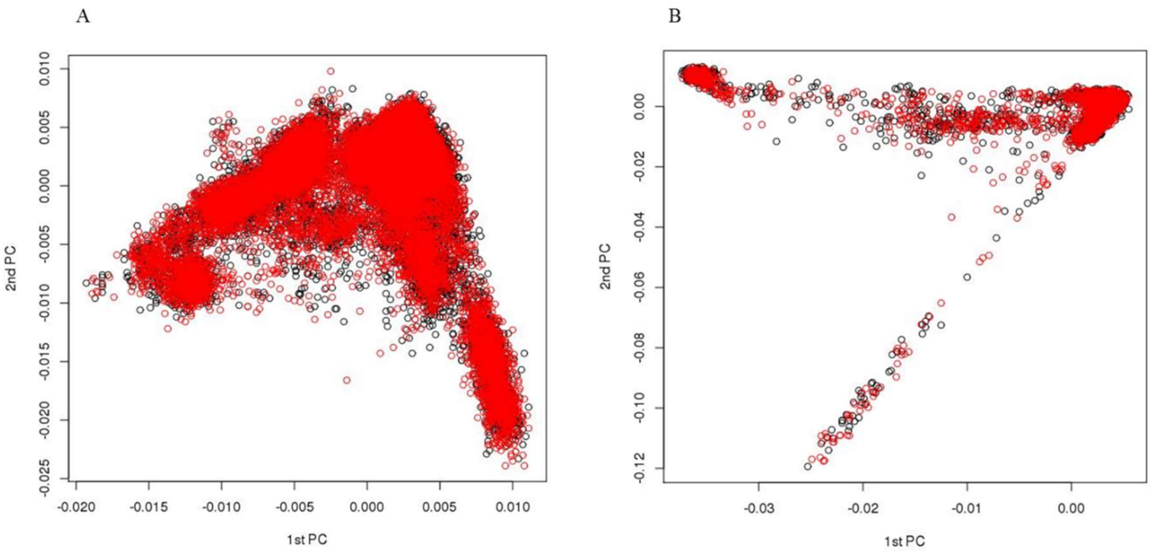

Supplementary Figure 4. Principal component (PC) analysis plot of first two principal components. The 1st PC axis is the first principal direction along which the samples show the largest variation. The 2nd PC axis is the second most important direction and it is orthogonal to the 1st PC axis. Each sample (dot) is color-coded by case or control status with control being black and case being red. **A,** the discovery dataset. For 1st PC and 2nd PC, the P value of anova statistics for population differences between Control and Case are 2.18E-05 and 0.0001, respectively. **B,** the replication dataset. For 1st PC and 2nd PC, the P value of anova statistics for population differences between Control and Case are 0.287 and 0.189, respectively.

Supplementary table 1. Participant characteristics of lung cancer cases and controls in study cohorts

Variants	Discovery Dataset (28878)					Replication Dataset (n =10268)				
	Control (n = 13027)		Case (n = 15851)			Control (n = 5352)		Case (n = 4916)		
	No.	%	No.	%	*P* -value	No.	%	No.	%	*P* -value
Age (years)										
<65	7727	59.32	8100	51.1	<.0001	2594	48.47	2064	41.99	<.0001
>=65	5251	40.31	7732	48.78		1415	26.44	1767	35.94	
missing	49	0.38	19	0.12		1343	25.09	1085	22.07	
Gender										
Male	7922	60.81	10027	63.26	<.0001	2870	53.62	2671	54.33	0.33
Female	5102	39.16	5819	36.71		2481	46.36	2222	45.2	
missing	3	0.02	5	0.03		1	0.02	23	0.47	
Smoking Status										
Never	3942	30.26	1460	9.21	<.0001	1597	29.84	587	11.94	<.0001
Ever	8748	67.15	14019	88.44		3736	69.81	4291	87.29	
missing	337	2.59	372	2.35		19	0.36	38	0.77	
Histology										
Squamous			3830	24.16				1073	21.83	
Adenocarcinoma			6334	39.96				2161	43.96	
Small cell carcinoma			1723	10.87				533	10.84	
Other/ Missing			3964	25.01				1149	23.37	

Supplementary table 2. The 3 germline mutations affecting lung cancer risk with high effect with association *P* value of less than 5.0 x 10^{-8} and OR value of more than 2.0 in the discovery dataset

						Discovery Dataset						Replication Dataset					
CHR	SNP	BP*	Gene	Area	function	Minor Allele	Major Allele	F_A[a]	F_U[b]	OR (95% CI)	*P*-value	Minor Allele	Major Allele	F_A	F_U	OR (95% CI)	*P*-value
1	rs61816761	152285861	*FLG*	exonic	stop	G	A	2.02E-03	2.73E-04	7.43 (3.41 - 16.22)	3.30E-09	A	G	1.47E-02	1.82E-02	0.81 (0.65 - 1.00)	0.05
11	rs56009889	108196896	*ATM*	exonic	Leu2307Phe	T	C	2.75E-03	6.91E-04	3.98 (2.40 - 6.61)	8.21E-09	T	C	1.94E-03	1.40E-03	1.39 (0.70 - 2.73)	0.34
22	rs150665432	45608215	*KIAA0930*	exonic	stop	A	G	1.36E-02	4.94E-03	2.78 (2.27 - 3.39)	1.71E-25	A	G	4.61E-03	3.00E-03	1.54 (0.98 - 2.42)	0.06

*Physical positions are based on NCBI build 37 of the human genome.

[a]Frequency of this allele in cases (F_A)

[b]Frequency of this allele in controls (F_U)

Supplementary table 3. Concordance table for 5742 individuals who are overlapped between Oncoarray and Affymetrix for rs61816761, rs56009889 and rs150665432

SNP	OncoArray ID	Affymetrix ID	General Concordance	Concordance of Rare Allele
rs61816761	rs61816761	AX-88734143	0%	0%
rs56009889	chr11_108196896_C_T	AX-83007922	99.95%	89.66%
rs150665432	chr22_45608215_A_G	AX-83492389	99.08%	92.31%

Supplementary table 4. Minor Allele Frequencies (MAFs) of unaffected individuals in study datasets being in agreement with those in public sequencing projects for rs61816761, rs56009889 and rs150665432

							ExAC[b]										
			Discovery Dataset		Replication Dataset		European (Non-Finnish)				European (Finnish)			GO-ESP[c]		TOPMED[d]	
CHR	Rare variants	BP	MAF	Minor Allele	MAF	Minor Allele	RAC[a]	Minor Allele	All Allele Number	MAF	RAC[a]	Minor Allele	MAF	MAF	Minor Allele	MAF	Minor Allele
11	rs56009889	108196896	6.91E-04	T	1.40E-03	T	129	T	66722	1.93E-03	1	T	1.51E-04	1.80E-03	T	7.00E-04	T
22	rs150665432	45608215	4.94E-03	A	3.00E-03	A	375	A	63792	5.88E-03	16	A	2.43E-03	2.90E-03	A	3.30E-03	A

[a]RAC is rare allele count

[b]the Exome Aggregation Consortium (ExAC)

[c]NHLBI GO Exome Sequencing Project (GO-ESP)

[d]Trans-Omics for Precision Medicine Program (TOPMed)

Supplementary table 5. Stratification of association between rs56009889 (*ATM*- L2307F) and risk of lung cancer by age, gender, smoking status and histology of lung cancer

			Discovery Dataset												Replication Dataset											
			Control		Case		Crude				Adjusted[a]				Control		Case		Crude				Adjusted[a]			
Group	Outcome	Genotype	No.	%	No.	%	OR (95%CI)			*P* -value	OR (95%CI)			*P* -value	No.	%	No.	%	OR (95%CI)			*P* -value	OR (95%CI)			*P* -value
All	Lung cancer	CC	13005	99.86	15767	99.48	1.00				1.00				5331	99.72	4891	99.61	1.00				1.00			
		TC	18	0.14	77	0.49	3.528	2.11	5.90	1.50E-06	3.79	2.17	6.60	2.57E-06	15	0.28	19	0.39	1.38	0.70	2.72	0.35	1.31	0.65	2.65	0.45
		TT	0	0	5	0.03	Inf	0.76	Inf	0.068*	-	-	-	-	0	0	0	0	-	-	-	-	-	-	-	-
		TC+TT	18	0.14	82	0.52	3.76	2.26	6.26	3.77E-07	4.19	2.41	7.28	3.56E-07	15	0.28	19	0.39	1.38	0.70	2.72	0.35	1.31	0.65	2.65	0.45
		Trend								3.67E-07				2.45E-07					1.38	0.70	2.72	0.35				
Female	Lung cancer	CC	5096	99.92	5777	99.28	1.00				1.00				2475	99.8	2203	99.32	1.00				1.00			
		TC	4	0.08	41	0.7	9.04	3.24	25.26	2.66E-05	7.67	2.64	22.28	0.0002	5	0.2	15	0.68	3.36	1.22	9.26	0.02	3.22	1.12	9.21	0.03
		TT	0	0	1	0.02	Inf	0.03	Inf	0.49*	-	-	-	-	0	0	0	0	-	-	-	-	-	-	-	-
		TC+TT	4	0.08	42	0.72	9.26	3.32	25.85	2.13E-05	7.76	2.68	22.49	0.0002	5	0.2	15	0.68	3.36	1.22	9.26	0.02	3.22	1.12	9.21	0.03
		Trend								2.38E-05				0.0002												
Male	Lung cancer	CC	7906	99.82	9985	99.6	1.00								2855	99.65	2665	99.85	1.00				1.00			
		TC	14	0.18	36	0.36	2.04	1.10	3.78	0.02	2.72	1.35	5.48	0.005	10	0.35	4	0.15	0.43	0.13	1.37	0.15	0.40	0.12	1.31	0.13
		TT	0	0	4	0.04	Inf	0.52	Inf	0.14*	-	-	-	-	0	0	0	0	-	-	-	-	-	-	-	-
		TC+TT	14	0.18	40	0.4	2.26	1.23	4.16	0.009	3.34	1.68	6.66	0.0006	10	0.35	4	0.15	0.43	0.13	1.37	0.15	0.40	0.12	1.31	0.13
		Trend								0.006				0.0002												
Non-smok	Lung cancer	CC	3933	99.8	1434	98.22	1.00				1.00				1589	99.69	581	99.32					1.00			
		TC	8	0.2	23	1.58	7.88	3.52	17.66	5.27E-07	4.31	1.86	10.00	0.0007	5	0.31	4	0.68	2.19	0.59	8.18	0.24	1.78	0.47	6.77	0.39
		TT	0	0	3	0.21	Inf	1.13	Inf	0.02*	-	-	-	-	0	0	0	0	-	-	-	-	-	-	-	-
		TC+TT	8	0.2	26	1.78	8.91	4.03	19.73	6.86E-08	5.00	2.18	11.44	0.0001	5	0.31	4	0.68	2.19	0.59	8.18	0.24	1.78	0.47	6.77	0.39
		Trend								1.46E-07				0.0001												
Smoker	Lung cancer	CC	8735	99.89	13963	99.61	1.00				1.00				3723	99.73	4273	99.67	1.00				1.00			
		TC	10	0.11	52	0.37	3.25	1.65	6.40	6.00E-04	2.76	1.38	5.51	0.004	10	0.27	14	0.33	1.22	0.54	2.74	0.64	1.07	0.47	2.45	0.87
		TT	0	0	2	0.01	Inf	0.12	Inf	0.53*	-	-	-	-	0	0	0	0	-	-	-	-	-	-	-	-
		TC+TT	10	0.11	54	0.39	3.38	1.72	6.64	0.0004	2.85	1.43	5.69	0.003	10	0.27	14	0.33	1.22	0.54	2.74	0.64	1.07	0.47	2.45	0.87
		Trend								0.0004				0.003												
Overall	Adenocarcin	CC	13005	99.86	6267	98.96	1.00				1.00				5331	99.72	2139	99.17	1.00				1.00			
		TC	18	0.14	61	0.96	7.019	4.15	11.88	3.97E-13	4.68	2.66	8.22	7.92E-08	15	0.28	18	0.83	2.98	1.50	5.93	0.002	2.48	1.22	5.04	0.01
		TT	0	0	5	0.08	Inf	1.90	Inf	0.004*	-	-	-	-	0	0	0	0	-	-	-	-	-	-	-	-
		TC+TT	18	0.14	66	1.04	7.60	4.51	12.81	2.59E-14	5.23	2.99	9.15	6.47E-09	15	0.28	18	0.83	2.98	1.50	5.93	0.002	2.48	1.22	5.04	0.01
		Trend								6.38E-14				5.44E-09												
Overall	Squamous ce	CC	13005	99.86	3827	99.92	1.00								5331	99.72	1073	100	1.00				1.00			
		TC	18	0.14	3	0.08	0.57	0.17	1.93	0.36	0.81	0.22	2.99	0.75	15	0.28	0	0	0	0	1.39	0.15*	-	-	-	-
		TT	0	0	0	0	-	-	-	-	-	-	-	-	0	0	0	0	-	-	-	-	-	-	-	-
		TC+TT	18	0.14	3	0.08	0.57	0.17	1.93	0.36	0.81	0.22	2.99	0.75	15	0.28	0	0	0	0	1.39	0.15*	-	-	-	-
		Trend												0.75												
Overall	Small cell lu	CC	13005	99.86	1721	99.88	1.00				1.00				5331	99.72	532	100	1.00				1.00			
		TC	18	0.14	2	0.12	0.84	0.20	3.62	0.81	2.09	0.44	9.87	0.35	15	0.28	0	0	0	0	2.80	0.39*	-	-	-	-
		TT	0	0	0	0	-	-	-	-	-	-	-	-	0	0	0	0	-	-	-	-	-	-	-	-
		TC+TT	18	0.14	2	0.12	0.84	0.20	3.62	0.81	2.09	0.44	9.87	0.35	15	0.28	0	0	0	0	2.80	0.39	-	-	-	-
		Trend												0.35												

[a]Adjusted for age at diagnosis/interview, gender, smoking status and PCs.

*Values generated from two-sided Fisher's Exact Test.

Supplementary table 6. Association between rs56009889 (*ATM* -L2307F) and risk of lung adenocarcinoma in female, non-smokers and the subgroup of female non-smokers

		**Discovery Dataset**												**Replication Dataset**											
		Control		Case		Crude				Adjusted[a]				Control		Case		Crude				Adjusted[a]			
	Genotype	No.	%	No.	%	OR (95%CI)			*P* -value	OR (95%CI)			*P* -value	No.	%	No.	%	OR (95%CI)			*P* -value	OR (95%CI)			*P* -value
Non-smoker	CC	3933	99.8	908	97.53	1.00				1.00				1589	99.69	373	99.2	1.00				1.00			
	TC	8	0.2	20	2.15	10.83	4.75	24.66	1.41E-08	5.05	2.11	12.11	3.00E-04	5	0.31	3	0.8	2.56	0.61	10.74	0.20	1.82	0.42	7.88	0.42
	TT	0	0	3	0.32	Inf	1.79	Inf	6.63E-03*	-	-	-	-	0	0	0	0	-	-	-	-	-	-	-	-
	TC+TT	8	0.2	23	2.47	12.45	5.55	27.92	9.40E-10	6.03	2.56	14.19	3.96E-05	5	0.31	3	0.8	2.56	0.61	10.74	0.20	1.82	0.42	7.88	0.42
	Trend								3.51E-09				4.29E-05												
Female	CC	5096	99.92	2923	98.88	1.00				1.00				2475	99.8	1186	98.83	1.00				1.00			
	TC	4	0.08	32	1.08	13.94	4.93	39.43	6.89E-07	7.91	2.71	23.11	0.0002	5	0.2	14	1.17	5.84	2.10	16.26	0.0007	4.69	1.65	13.35	0.004
	TT	0	0	1	0.04	Inf	0.04	Inf	0.36*	-	-	-	-	0	0	0	0	-	-	-	-	-	-	-	-
	TC+TT	4	0.08	33	1.12	14.37	5.09	40.58	4.88E-07	8.05	2.76	23.47	0.0001	5	0.2	14	1.17	5.84	2.10	16.26	0.0007	4.69	1.65	13.35	0.004
	Trend								6.08E-07				0.0001												
Female non-smoker	CC	2153	99.91	651	98.34	1.00				1.00				937	99.79	270	98.9	1.00				1.00			
	TC	2	0.09	11	1.66	18.19	4.02	82.27	0.0002	7.09	1.52	33.09	0.01	2	0.21	3	1.1	5.20	0.87	31.31	0.07	3.80	0.61	23.72	0.15
	TT	0	0	0	0	-	-	-	-	-	-	-	-	0	0	0	0	-	-	-	-	-	-	-	-
	TC+TT	2	0.09	11	1.66	18.19	4.02	82.27	0.0002	7.09	1.52	33.09	0.01	2	0.21	3	1.1	5.20	0.87	31.31	0.07	3.80	0.61	23.72	0.15
	Trend																								

[a]Adjusted for age at diagnosis/interview, gender, smoking status and PCs.

*Values generated from two-sided Fisher's Exact Test.

Supplementary table 7. In the discovery dataset, association between rs56009889 (*ATM* -L2307F) and risk of lung cancer and of lung adenocarcinoma in overall and in females in different countries

Geographic populations	Group	Outcome	Genotype	Control		Case		Crude				Adjusted[a]			
				No.	%	No.	%	OR (95%CI)			*P* -value	OR (95%CI)			*P* -value
North Americans	All	Lung cancer	CC	4132	99.78	6679	99.43	1.00				1.00			
			TC	9	0.22	36	0.54	2.48	1.19	5.14	0.02	2.61	1.17	5.80	0.02
			TT	0	0	2	0.03	Inf	0.116	Inf	0.53*	-	-	-	-
			TC+TT	9	0.22	38	0.57	2.61	1.26	5.41	0.01	2.68	1.21	5.95	0.02
			Trend								0.009				0.01
Israeli	All	Lung cancer	CC	502	98.43	619	93.36	1.00				1.00			
			TC	8	1.57	41	6.18	4.16	1.93	8.95	0.0003	4.65	2.10	10.32	0.0002
			TT	0	0	3	0.45	Inf	0.334	Inf	0.26*	-	-	-	-
			TC+TT	8	1.57	44	6.64	4.46	2.08	9.56	0.0001	5.32	2.41	11.78	3.72E-05
			Trend								0.0001				3.51E-05
Population in other European countries	All	Lung cancer	CC	8371	99.99	8469	100	1.00				1.00			
			TC	1	0.01	0	0	0.00	0.00	38.55	0.50*	-	-	-	-
			TT	0	0	0	0	-	-	-	-	-	-	-	-
			TC+TT	1	0.01	0	0	0.00	0	38.55	0.50*	-	-	-	-
			Trend												-
North Americans	All	LAD	CC	4132	99.78	3035	99.05								
			TC	9	0.22	27	0.88	4.08	1.92	8.69	0.0003	3.21	1.40	7.34	0.006
			TT	0	0	2	0.07	Inf	0.26	Inf	0.18*	-	-	-	-
			TC+TT	9	0.22	29	0.95	4.38	2.07	9.26	0.0001	3.36	1.48	7.62	0.004
			Trend								0.0001				0.004
Israeli	All	LAD	CC	502	98.43	375	91.02								
			TC	8	1.57	34	8.25	5.69	2.60	12.43	1.31E-05	5.87	2.62	13.18	1.77E-05
			TT	0	0	3	0.73	Inf	0.55	Inf	0.08*	-	-	-	-
			TC+TT	8	1.57	37	8.98	6.19	2.85	13.45	4.11E-06	6.74	3.01	15.08	3.38E-06
			Trend								5.44E-06				3.85E-06
North Americans	Female	LAD	CC	1985	99.85	1635	99.03								
			TC	3	0.15	15	0.91	6.07	1.75	21.00	0.0044	3.64	1.00	13.24	0.05
			TT	0	0	1	0.06	Inf	0.03	Inf	0.45*	-	-	-	-
			TC+TT	3	0.15	16	0.97	6.47	1.88	22.25	0.003	3.81	1.05	13.76	0.04
			Trend								0.003				0.04
Israeli	Female	LAD	CC	187	99.47	168	90.81								
			TC	1	0.53	17	9.19	18.91	2.49	143.49	0.005	17.15	2.24	131.32	0.006
			TT	0	0	0	0	-	-	-	-	-	-	-	-
			TC+TT	1	0.53	17	9.19	18.91	2.49	143.49	0.005	17.15	2.24	131.32	0.006
			Trend								-				-

[a]Adjusted for age at diagnosis/interview, gender, smoking status and PCs.

*Values generated from two-sided Fisher's Exact Test.

Supplementary table 8. In the discovery dataset, association between rs56009889 (*ATM* -L2307F) and risk of lung cancer and of lung adenocarcinoma in Jews and Arabs

			Control		Case		Crude				Adjusted[a]			
Ethnic group	Outcome	Genotype	No.	%	No.	%	OR (95%CI)			*P* -value	OR (95%CI)			*P* -value
Jews	Lung cancer	CC	428	96.61	620	88.7	1.00							
		TC	15	3.39	74	10.59	3.41	1.93	6.01	2.39E-05	3.80	2.11	6.83	8.39E-06
		TT	0	0	5	0.72	Inf	0.63	Inf	0.08*	-	-	-	-
		TC+TT	15	3.39	79	11.3	3.64	2.07	6.40	7.72E-06	4.18	2.33	7.49	1.66E-06
		Trend									4.05	2.30	7.13	1.32E-06
Arab	Lung cancer	CC	295	100	413	99.76	1.00							
		TC	0	1.57	1	0.24	Inf	0.02	Inf	1.00*	-	-	-	-
		TT	0	0	0	0	-	-	-	-	-	-	-	-
		TC+TT	0	1.57	1	0.24	Inf	0.02	Inf	1.00*	-	-	-	-
		Trend								-				-
Jews	LAD	CC	428	96.61	378	85.52	1.00							
		TC	15	3.39	59	13.35	4.45	2.49	7.98	5.21E-07	4.85	2.66	8.84	2.51E-07
		TT	0	0	5	1.13	Inf	1.03	Inf	0.02*	-	-	-	-
		TC+TT	15	3.39	64	14.48	4.83	2.71	8.62	9.74E-08	5.39	2.97	9.79	3.07E-08
		Trend								1.12E-07				3.17E-08

[a]Adjusted for age at diagnosis/interview, gender, smoking status and PCs.

*Values generated from two-sided Fisher's Exact Test.

Supplementary table 9. In the discovery dataset, association between rs56009889 (*ATM* -L2307F) and risk of lung cancer and of lung adenocarcinoma in overall and in females in Jews among different countries

Geographic populations	Groups	Outcome	Genotype	Control		Case		Crude				Adjusted[a]			
				No.	%	No.	%	OR (95%CI)			*P* -value	OR (95%CI)			*P* -value
Israeli	Jews	Lung cancer	CC	256	97.34	320	88.15	1.00				1.00			
			TC	7	2.66	40	11.02	4.57	2.01	10.37	0.0003	5.29	2.29	12.23	9.89E-05
			TT	0	0	3	0.83	Inf	0.33	Inf	0.26*	-	-	-	-
			TC+TT	7	2.66	43	11.85	4.91	2.17	11.10	0.0001	6.02	2.60	13.90	2.68E-05
			Trend								0.0001				2.86E-05
non-Israeli	Jews	Lung cancer	CC	172	95.56	300	89.29	1.00				1.00			
			TC	8	4.44	34	10.12	2.44	1.10	5.38	0.03	2.51	1.09	5.75	0.03
			TT	0	0	2	0.6	Inf	0.11	Inf	0.54*	-	-	-	-
			TC+TT	8	4.44	36	10.71	2.58	1.17	5.68	0.02	2.61	1.14	5.94	0.02
			Trend								0.02				0.02
Israeli	Jews	LAD	CC	256	97.34	202	84.87	1.00				1.00			
			TC	7	2.66	33	13.87	5.97	2.59	13.79	2.79E-05	6.90	2.93	16.23	9.68E-06
			TT	0	0	3	1.26	Inf	0.52	Inf	0.09*	-	-	-	-
			TC+TT					6.52	2.84	14.95	9.67E-06	7.86	3.35	18.44	2.12E-06
			Trend								1.28E-05				2.83E-06
non-Israeli	Jews	LAD	CC	172	95.56	176	86.27	1.00				1.00			
			TC	8	4.44	26	12.75	3.18	1.40	7.21	0.006	3.20	1.36	7.56	0.01
			TT	0	0	2	0.98	Inf	0.18	Inf	0.50*	-	-	-	-
			TC+TT	8	4.44	28	13.73	3.42	1.52	7.72	0.003	3.40	1.45	7.95	0.005
			Trend								0.003				0.005
Israeli	emale Jev	LAD	CC	112	99.12	115	87.79	1.00				1.000			
			TC	1	0.88	16	12.21	15.57	2.03	119.35	0.008	16.01	2.07	123.61	0.008
			TT	0	0	0	0	-	-	-	-	-	-	-	-
			TC+TT	1	0.88	16	12.21	15.57	2.03	119.35	0.008	16.01	2.07	123.61	0.008
			Trend								-				-
non-Israeli	emale Jev	LAD	CC	85	96.59	93	86.11	1.00				1.00			
			TC	3	3.41	14	12.96	4.27	1.18	15.36	0.03	3.97	1.05	14.97	0.04
			TT	0	0	1	0.93	Inf	0.02	Inf	1.00*				
			TC+TT	3	3.41	15	13.89	4.57	1.28	16.34	0.02	4.23	1.13	15.80	0.03
			Trend								0.02				0.03

[a]Adjusted for age at diagnosis/interview, gender, smoking status and PCs.

*Values generated from two-sided Fisher's Exact Test.

Supplementary table 10. Stratification of association between rs150665432 (*KIAA0930* -Q4X) and risk of lung cancer by gender and smoking status

		Discovery Dataset												Replication Dataset											
		Control		Case		Crude				Adjusted[a]				Control		Case		Crude				Adjusted[a]			
	Genotype	No.	%	No.	%	OR (95%CI)			*P* -value	OR (95%CI)			*P* -value	No.	%	No.	%	OR (95%CI)			*P* -value	OR (95%CI)			*P* -value
All	GG	12642	99.01	14814	97.47	1.00				1.00				5308	99.4	4861	99.04	1.00				1.00			
	AG	126	0.99	355	2.34	2.40	1.96	2.95	4.75E-17	2.41	1.95	2.99	7.83E-16	32	0.31	47	0.46	1.60	1.02	2.52	0.04	1.69	1.05	2.70	0.03
	AA	0	0	29	0.19	Inf	6.29	Inf	2.29E-08*	-	-	-	-	0	0	0	0	-	-	-	-	-	-	-	-
	AG+AA	126	0.99	384	2.53	2.60	2.12	3.19	2.34E-20	2.59	2.10	3.20	1.15E-18	32	0.31	47	0.46	1.60	1.02	2.52	0.04	1.69	1.05	2.70	0.03
	Trend								2.06E-21				1.51E-19												-
Femal	GG	4984	99.16	5539	97.66	1.00				1.00				2461	99.39	2199	99.14	1.00				1.00			
	AG	42	0.84	119	2.1	2.55	1.79	3.63	2.25E-07	2.59	1.78	3.75	5.92E-07	15	0.61	19	0.86	1.42	0.72	2.80	0.31	1.57	0.77	3.21	0.22
	AA	0	0	14	0.25	Inf	2.98	Inf	0.0002*	-	-	-	-	0	0	0	0	-	-	-	-	-	-	-	-
	AG+AA	42	0.84	133	2.34	2.85	2.01	4.04	4.10E-09	2.86	1.98	4.12	2.06E-08	15	0.61	19	0.86	1.42	0.72	2.80	0.31	1.57	0.77	3.21	0.22
	Trend								1.47E-09				9.64E-09												-
Male	GG	7655	98.91	9270	97.36	1.00				1.00				2846	99.41	2641	99.03	1.00				1.00			
	AG	84	1.09	236	2.48	2.32	1.80	2.98	5.11E-11	2.31	1.78	3.01	4.01E-10	17	0.59	26	0.97	1.65	0.89	3.04	0.11	1.65	0.87	3.12	0.12
	AA	0	0	15	0.16	Inf	2.96	Inf	1.27E-04*	-	-	-	-	0	0	0	0	-	-	-	-	-	-	-	-
	AG+AA	84	1.09	251	2.64	2.47	1.92	3.17	1.14E-12	2.45	1.89	3.18	1.44E-11	17	0.59	26	0.97	1.65	0.89	3.04	0.11	1.65	0.87	3.12	0.12
	Trend								2.95E-13				4.17E-12												-
Non-smoker	GG	3847	98.95	1389	97.47	1.00				1.00				1585	99.31	578	98.97	1.00				1.00			
	AG	41	1.05	34	2.39	2.30	1.45	3.64	0.0004	2.37	1.49	3.77	3.00E-04	11	0.69	6	1.03	1.50	0.55	4.06	0.43	1.56	0.57	4.29	0.39
	AA	0	0	2	0.14	Inf	0.52	Inf	0.07*	-	-	-	-	0	0	0	0	-	-	-	-	-	-	-	-
	AG+AA	41	1.05	36	2.53	2.43	1.55	3.82	0.0001	2.51	1.59	3.97	8.20E-05	11	0.69	6	1.03	1.50	0.55	4.06	0.43	1.56	0.57	4.29	0.39
	Trend								5.72E-05				3.82E-05												-
Smoker	GG	8465	99.06	13058	97.41	1.00				1.00				3704	99.44	4246	99.07	1.00				1.00			
	AG	80	0.94	320	2.39	2.59	2.03	3.32	3.58E-14	2.56	2.00	3.28	1.18E-13	21	0.26	40	0.5	1.66	0.98	2.82	0.06	1.65	0.97	2.82	0.07
	AA	0	0	27	0.2	Inf	4.427	Inf	2.61E-06*	-	-	-	-	0	0	0	0	-	-	-	-	-	-	-	-
	AG+AA	80	0.94	347	2.59	2.81	2.20	3.59	1.20E-16	2.77	2.16	3.54	5.34E-16	21	0.26	40	0.5	1.66	0.98	2.82	0.06	1.65	0.97	2.82	0.07
	Trend								3.33E-17				1.54E-16												-

[a]Adjusted for age at diagnosis/interview, gender, smoking status and PCs.

*Values generated from two-sided Fisher's Exact Test.

Supplementary table 11. Association between rs150665432 (*KIAA0930* -Q4X) and risk of lung adenocarcinoma, small cell lung cancer and squamous cell carcinoma in overall, smokers and male smokers

			Discovery Dataset												Replication Dataset											
			Control		Case		Crude				Adjusted[a]				Control		Case		Crude				Adjusted[a]			
Group	Outcome	Genotype	No.	%	No.	%	OR (95%CI)			*P* -value	OR (95%CI)			*P* -value	No.	%	No.	%	OR (95%CI)			*P* -value	OR (95%CI)			*P* -value
Overall	Adenocarcinoma	GG	12642	99.01	6019	97.74					1.00				5308	99.4	2136	98.98	1.00				1.00			
		AG	126	0.99	129	2.09	2.15	1.679	2.754	1.32E-09	2.37	1.83	3.07	4.91E-11	32	0.6	22	1.02	1.71	0.99	2.95	0.05	1.75	0.99	3.09	0.054
		AA	0	0	10	0.16	Inf	4.705	Inf	1.23E-05*	-	-	-	-	0	0	0	0	-	-	-	-	-	-	-	-
		AG+AA	126	0.99	139	2.26	2.316	1.817	2.954	1.25E-11	2.52	1.96	3.25	7.66E-13	32	0.6	22	1.02	1.71	0.99	2.95	0.05	1.75	0.99	3.09	0.054
		Trend								8.31E-13				1.39E-13								-				-
Overall	Small cell lung cancer	GG	12642	99.01	1593	97.25	1.00				1.00				5308	99.4	525	98.87	1.00				1.00			
		AG	126	0.99	41	2.5	2.58	1.81	3.69	1.78E-07	2.63	1.81	3.82	3.99E-07	32	0.6	6	1.13	1.90	0.79	4.56	0.15	1.95	0.78	4.85	0.15
		AA	0	0	4	0.24	Inf	5.23	Inf	1.58E-04*	-	-	-	-	-	0	0	0	-	-	-	-	-	-	-	-
		AG+AA	126	0.99	45	2.75	2.84	2.01	4.00	3.00E-09	2.87	2.00	4.13	1.11E-08	32	0.6	6	1.13	1.90	0.79	4.56	0.15	1.95	0.78	4.85	0.15
		Trend								1.31E-10				1.22E-09								-				-
Overall	Squamous cell carcinoma	GG	12642	99.01	3554	97.34	1.00				1.00				5308	99.4	1061	99.07	1.00				1.00			
		AG	126	0.99	89	2.44	2.51	1.91	3.30	4.34E-11	2.36	1.75	3.18	1.84E-08	32	0.6	10	0.93	1.57	0.77	3.19	0.22	1.56	0.74	3.31	0.25
		AA	0	0	8	0.22	Inf	6.07	Inf	5.42E-06*	-	-	-	-	0	0	0	0	-	-	-	-	-	-	-	-
		AG+AA	126	0.99	97	2.66	2.74	2.10	3.58	1.52E-13	2.59	1.93	3.47	2.05E-10	32	0.6	10	0.93	1.57	0.77	3.19	0.22	1.56	0.74	3.31	0.25
		Trend								5.90E-15				1.41E-11								-				-
Smoker	Adenocarcinoma	GG	8465	99.06	4992	97.84	1.00				1.00				3704	99.44	1742	98.98	1.00				1.00			
		AG	80	0.94	100	1.96	2.12	1.58	2.85	6.60E-07	2.22	1.64	3.00	2.46E-07	21	0.56	18	1.02	1.82	0.97	3.43	0.06	1.73	0.90	3.30	0.10
		AA	0	0	10	0.2	Inf	3.80	Inf	4.97E-05*	-	-	-	-	0	0	0	0	-	-	-	-	-	-	-	-
		AG+AA	80	0.94	110	2.16	2.33	1.74	3.12	1.08E-08	2.42	1.80	3.26	4.93E-09	21	0.56	18	1.02	1.82	0.97	3.43	0.06	1.73	0.90	3.30	0.10
		Trend								1.19E-09				8.02E-10								-				-
Smoker	Small cell lung cancer	GG	8465	99.06	1502	97.09	1.00				1.00				3704	99.44	503	99.02	1.00				1.00			
		AG	80	0.94	41	2.65	2.89	1.97	4.23	4.63E-08	2.86	1.95	4.19	7.02E-08	21	0.56	5	0.98	1.75	0.66	4.67	0.26	1.70	0.63	4.56	0.30
		AA	0	0	4	0.26	Inf	3.72	Inf	5.19E-04*	-	-	-	-	0	0	0	0	-	-	-	-	-	-	-	-
		AG+AA	80	0.94	45	2.91	3.17	2.19	4.59	9.17E-01	3.13	2.16	4.54	1.57E-09	21	0.56	5	0.98	1.75	0.66	4.67	0.26	1.70	0.63	4.56	0.30
		Trend								9.26E-11				1.85E-10								-				-
Smoker	Squamous cell carcinoma	GG	8465	99.06	3376	97.26	1.00				1.00				3704	99.44	990	99	1.00							
		AG	80	0.94	88	2.54	2.76	2.03	3.74	7.79E-11	2.52	1.85	3.44	5.05E-09	21	0.56	10	1	1.78	0.84	3.80	0.13	1.72	0.79	3.72	0.17
		AA	0	0	7	0.2	Inf	3.61	Inf	1.54E-04*	-	-	-	-	0	0	0	0	-	-	-	-	-	-	-	-
		AG+AA	80	0.94	95	2.74	2.98	2.21	4.02	1.03E-12	2.72	2.01	3.69	1.23E-10	21	0.56	10	1	1.78	0.84	3.80	0.13	1.72	0.79	3.72	0.17
		Trend								1.76E-13				2.30E-11								-				-

[a]Adjusted for age at diagnosis/interview, gender, smoking status and PCs.

*Values generated from two-sided Fisher's Exact Test.

Supplementary table 12. Association between rs150665432 (*KIAA0930* -Q4X) and risk of lung cancer in different geographic populations in the discovery dataset

Geographic Populations	Group	Outcome	Genotype	Control		Case		Crude				Adjusted[a]			
				No.	%	No.	%	OR (95%CI)			*P* -value	OR (95%CI)			*P* -value
North Americans	All	Lung cancer	GG	3998	99.18	6213	96.34								
			AG	33	0.82	207	3.21	4.04	2.79	5.84	1.36E-13	3.66	2.50	5.37	3.1463E-11
			AA	0	0	29	0.45	Inf	4.74	Inf	7.83E-07*	-	-	-	-
			AG+AA	33	0.82	236	3.66	4.60	3.19	6.63	3.27E-16	4.19	2.87	6.12	1.2154E-13
			Trend								6.31E-16				1.60847E-13
Population in European countries	All	Lung cancer	GG	8644	98.94	8601	98.31								
			AG	93	1.06	148	1.69	1.60	1.23	2.08	0.0004	1.65	1.26	2.18	0.0003
			AA	0	0	0	0	-	-	-	-	-	-	-	-
			AG+AA	93	1.06	148	1.69	1.60	1.23	2.08	0.0004	1.65	1.26	2.18	0.0003
			Trend								0.0004				0.0003

[a]Adjusted for age at diagnosis/interview, gender, smoking status and PCs.

*Values generated from two-sided Fisher's Exact Test.

Supplementary table 13. Comparison of the onset time of lung cancer between wild genotype (C/C) and the variant genotypes (T/C + T/T) of rs56009889 (*ATM* -L2307F)

	**Discovery Dataset**									**Replication Dataset**								
	CC				TC + TT					CC				TC +TT				
Stratification	N	Mean	Std Dev	Median	N	Mean	Std Dev	Median	*P* -value	N	Mean	Std Dev	Median	N	Mean	Std Dev	Median	*P* -value
all	15748	63.69	10.50	64.32	82	69.08	10.53	69.6	<.0001	3809	63.85	9.75	64	17	67	11.31	67.41	0.18
female	5768	63.14	11.04	64.00	42	68.79	11.2	71.03	0.001	1640	63.51	9.94	63.85	13	67.26	11.37	69.71	0.18
male	9979	64.01	10.16	64.71	40	69.39	9.90	68.1	0.0008	2146	64.1	9.58	64	4	66.18	12.79	62.77	0.67
never smoker	1433	63.57	12.31	64.91	26	68.23	11.68	68.8	0.06	446	63.71	11.68	65	4	70.05	15.14	72.35	0.28
smoker	13952	63.68	10.27	64.20	54	69.2	10.08	69.95	<.0001	3333	63.84	9.47	63.8	12	66.66	10.68	66.97	0.30
adenocarcinoma	6263	63.64	10.72	64.36	66	68.83	10.35	68.8	<.0001	1654	64.25	10.41	64.35	16	68.1	10.71	68.56	0.14
small cell carcinoma	1717	61.53	10.57	62.00	2	61.59	24.62	61.59	0.9934	408	63.76	8.22	63.8	-	-	-	-	-
squamous cell carcinoma	3825	64.89	9.71	65.80	3	71.73	12.2	70.3	0.22	922	64.45	8.75	64.5	-	-	-	-	-
adenocarcinoma + never smoker	908	63.41	12.21	64.85	23	67.94	12.08	68.8	0.08	290	64.45	11.76	66	3	76.9	7.88	72.40	0.07
adenocarcinoma+ female	2921	62.78	11.06	63.49	33	69.37	10.71	69.4	0.0007	894	63.69	10.31	64	12	68.74	10.49	70.20	0.09

Supplementary table 14. Comparison of the onset time of lung cancer between wild genotype (G/G) and the variant genotypes (A/G + A/A) of rs150665432 (*KIAA0930* -Q4X)

	**Discovery Dataset**									**Replication Dataset**								
	G/G				A/G + A/A					G/G				A/G + A/A				
	N	Mean	Std Dev	Median	N	Mean	Std Dev	Median	*P* -value	N	Mean	Std Dev	Median	N	Mean	Std Dev	Median	*P* -value
all	14796	63.66	10.59	64.3	384	66.25	8.92	67	<.0001	3786	63.85	9.76	64	38	64.47	10.08	63.35	0.70
female	5530	62.97	11.08	63.7	133	67.38	8.71	68	<.0001	1637	63.51	9.95	63.89	16	64.57	9.61	64.26	0.67
male	9265	64.07	10.27	64.97	251	65.65	8.99	66	0.02	2128	64.10	9.59	64	20	65.43	9.41	63.35	0.54
never smoker	1389	63.50	12.37	64.75	36	64.41	10.88	66.22	0.66	2163	61.90	9.69	61.25	17	61.65	10.74	62.4	0.92
smoker	13047	63.64	10.37	64.2	347	66.41	8.69	67	<.0001	3312	63.84	9.47	63.8	32	64.65	9.22	63.35	0.63
small cell carcinoma	1589	61.41	10.70	62	45	64.21	9.26	66	0.08	402	63.71	8.06	63.8	5	65.82	18.34	63.7	0.57
adenocarcinoma	6019	63.61	10.75	64.17	139	66.05	9.61	67	0.008	1655	64.26	10.41	64.4	16	66.01	9.57	66.38	0.50
squamous cell carcinoma	3553	64.90	9.82	66	97	68.70	7.56	68.1	0.0002	911	64.46	8.78	64.62	9	64.12	5.40	63	0.91

Supplementary table 15. Association between Germline *ATM* L2307F and clinical variables

	GermlineATM L2307F		
	Yes (n=63)	No (n=2064)	Chi-square p-value
Age (years)			
<=60	15	353	1
>60	33	796	
Gender			
Female	37	848	0.03
Male	13	606	
Smoking Status			
Never	18	244	0.01
Ever	30	905	
Pack-years for smokers			
Light smokers (pack-years <=5)	24	335	0.003
Heavy Smokers (pack-years >5)	24	807	
Oncogenic Driver Mutations			
TP53 – present	26	1135	0.04
TP53 – absent	37	929	
EGFR – present	20	327	0.001
EGFR- absent	43	1737	
KRAS- present	22	609	0.4
KRAS –absent	41	1455	

Supplementary table 16. *ATM* isoform expression in normal lung tissue from Gtex Data

	Min.	1st Qu.	Median	Mean	3rd Qu.	Max.	aa length of the produced protein	Protein	Effect of rs56009889 on isoforms
ENST00000278616.4	1	33.5	89	99.06	150	439	3056	protein coding	Leu2307Phe
ENST00000452508.2	34	869.5	1013	988	1152.5	1488	3056	protein coding	Leu2307Phe
ENST00000527805.1	1	1	1	1.206	1	7	1369	protein coding	no
ENST00000531525.2	2	55	87	100.9	129.5	359	155	protein coding	no
ENST00000527891.1	1	1	1	37.23	60	290	154	protein coding	no
ENST00000532931.1	1	1	1	5.82	1	108	93	protein coding	no
ENST00000601453.1	1	1	1	3.16	1	54	135	protein coding	no
ENST00000529588.1	1	1	1	4.59	1	71	77	Nonsense mediated decay	no
ENST00000527181.1	1	173.5	588	524.7	744.5	1247	0		no
ENST00000527389.1	1	1	42	59.21	90	286	0		no
ENST00000530958.1	8	37.5	56	61.62	80	176	0		no
ENST00000531957.1	1	19.5	59	77.97	110.5	389	0		no
ENST00000532765.1	1	1	1	1.33	1	33	0		no
ENST00000533526.1	1	1	1	7.16	1	175	0		no
ENST00000533690.1	1	23	45	58.38	82	240	0		non coding transcript exon variant
ENST00000533733.1	1	9.5	17	20.57	26	76	0		no
ENST00000533979.1	1	14	30	45.75	64.5	194	0		no
ENST00000534625.1	1	1	5	14.88	22.5	113	0		no
ENST00000419286.1	1	1	1	1.73	1	80	0		no
ENST00000524792.1	1	1	1	2.46	1	55	0		non coding transcript exon variant
ENST00000525012.1	1	1	1	1	1	1	0		no
ENST00000525056.1	1	68	131	159.3	241.5	404	0		no
ENST00000525178.1	1	1	1	23.82	1	399	0		no
ENST00000525537.1	1	1	23	34.21	48	174	0		no
ENST00000526567.1	1	21	43	53.63	76	232	0		no

Supplementary table 17. *ATM* isoform expression in normal lung tissue from Gemany Data

	Min.	1st Qu.	Median	Mean	3rd Qu.	Max.
ENST00000278616	0	37.4	250.1	251.1	388.2	605.8
ENST00000419286	0	0	0	0	0	0
ENST00000452508	513.8	770.9	1087	1012.2	1187.9	1498.2
ENST00000524792	0	0	0	0	0	0
ENST00000525012	0	0	0	0	0	0
ENST00000525056	0	0	0	0.70	1.22	2.56
ENST00000525178	0	0	4.52	4.60	9.08	9.49
ENST00000525537	0	0	0	0.44	0	2.66
ENST00000526567	0	0.33	1.95	2.35	3.87	5.92
ENST00000527181	4	21.15	30.09	31.56	46.72	54.73
ENST00000527389	0	0	0	1.00	1.57	3.88
ENST00000527805	0	0	0	0.38	0	2.25
ENST00000527891	0	0	3.215	4.27	8.76	9.65
ENST00000529588	0	0	0	0.20	0	1.22
ENST00000530958	0	8.45	11.64	10.85	15.35	17.84
ENST00000531525	1.11	2.04	5.64	4.98	7.54	8.48
ENST00000531957	0	0.01	0.35	1.99	3.85	6.34
ENST00000532765	0	0	0	0	0	0
ENST00000532931	0	0	0	0.2	0	1.2
ENST00000533526	0	0	0	0	0	0
ENST00000533690	1.6	6.31	9.63	11.92	16.53	26.63
ENST00000533733	0	0	0.54	0.89	1.1	3.14
ENST00000533979	0	0	1.13	1.34	2.453	3.25
ENST00000534625	0	0	0	0.21	0	1.25
ENST00000601453	0	0	0	0	0	0

Supplementary table 18. *KIAA0930* isoform expression level in normal lung tissue from Gtex Data

	Min.	1st Qu.	Median	Mean	3rd Qu.	Max.	aa length of the produced protein	Effect of rs150665432 on isoforms
ENST00000251993.7	1	66	158	617.8	966.5	2637	409	stop gained, responsible for Gln4Ter
ENST00000336156.5	74	183.5	653	561	752.5	1062	404	no
ENST00000391627.2	1	525	959	1349	2404	2857	370	no
ENST00000414854.1	1	1	1	11.73	1	272	136	no
ENST00000417906.1	1	1	1	2.337	1	48	84	NMD transcript variant
ENST00000492273.1	1	1	1	4.703	1	138	85	Stop_gain, responsible for
ENST00000496226.1	1	1	1	7.368	1	153	89	5 prime UTR variant
ENST00000423262.1	2	489	1266	1749	3140	3473	289	no
ENST00000424508.1	1	1	1	15.71	1	151	152	no
ENST00000440039.1	1	1	1	14.09	19	250	70	no
ENST00000443310.3	1	1	1	96.87	135	756	0	no
ENST00000474515.1	1	1	1	2.752	1	86	0	no
ENST00000483374.1	1	1	22	29.73	45	181	0	no
ENST00000486640.1	1	1	1	19.04	31	169	0	non coding transcript exon variant
ENST00000488038.1	1	123.5	1020	971.6	1552.5	2576	0	non coding transcript exon variant
ENST00000493003.1	1	1	1	5.197	1	135	0	no
ENST00000498418.1	1	74	132	175.4	271	661	0	no

Supplementary table 19. *KIAA0930* isoform expression level in normal lung tissue from Germany Data

	Min.	1st Qu.	Median	Mean	3rd Qu.	Max.
ENST00000251993	0	5.67	33.66	69.59	72.16	268.88
ENST00000336156	226.3	244.1	312	375	372.7	785
ENST00000391627	0	56.23	349.28	320.72	502.6	713.53
ENST00000414854	0	0	0	0	0	0
ENST00000417906	0	0	0	0	0	0
ENST00000423262	298.6	339.1	398.6	397.4	429.4	529.8
ENST00000424508	0	0	0	0.50	0	2.98
ENST00000440039	0	0	0	0	0	0
ENST00000443310	26.1	43.28	68.27	64.44	78.5	107.15
ENST00000474515	0	0	0	4.60	8.24	16.58
ENST00000483374	0	0	0	0	0	0
ENST00000486640	0	0	1.2	1.85	2.91	5.62
ENST00000488038	0	7.705	45.16	57.56	104.91	135.01
ENST00000492273	0	0	0	0	0	0
ENST00000493003	0	0	0	0	0	0
ENST00000496226	0	0	0	0.17	0	1
ENST00000498418	0	0.95	4.25	4.53	6.497	11.61

Supplementary table 20. Comparison of gene expression of *KIAA0930*, exon 2 and 5 of *ATM* between Lung cancer (in general and within each histologic type) and normal lung tissue with Harvard Data

	KIAA0930				Exon 2 of ATM				Exon 5 of ATM			
Std Dev	N	Mean	Std Dev	*P*-value*	N	Mean	Std Dev	*P*-value*	N	Mean	Std Dev	*P*-value*
Normal Lung Tissue	17	-24.56	17.92	-	17	-15.87	15.25	-	17	71.90	33.38	-
Lung Cancer	186	-8.14	18.21	0.0005	186	-3.69	19.43	0.01	186	83.76	32.46	0.15
Lung Adenocarcinoma	139	-7.79	18.05	0.0004	139	-3.09	17.33	0.004	139	83.97	31.69	0.14
Lung squamous cell carcinoma	21	-20.88	8.76	0.41	21	-19.90	8.50	0.31	21	62.15	20.40	0.27
Small Cell Lung Cancer	6	-9.10	21.18	0.097	6	3.01	34.15	0.08	6	92.84	42.48	0.23
Lung carcinoid	20	3.11	18.68	<.0001	20	7.13	26.06	0.003	20	102.30	33.81	0.01

*comparison of Lung cancer (in general and within each histologic type) to normal lung tissue

Supplementary table 21. Comparison of gene expression of *KIAA0930* and *ATM* between primary tumor tissue and normal tissue with TCGA Data

Primary Tumor	Cancer Types#	Tumor Cell Source	Number of Samples		KIAA0930		ATM
			Normal Tissue	Tumor Tissue	regulation direction in Tumor	*P* -value*	*P* -value*
lung adenocarcinoma (LUAD)	Carcinoma	glandular epithelium	59	515	up	0.004	0.08
lung squamous cell carcinoma (LUSC)	Carcinoma	Squamous epithelium	52	503	up	1.62E-12	0.08
breast invasive carcinoma (BRCA)	Carcinoma	glandular epithelium	114	1097	up	5.10E-07	1.11E-16
cervical squamous cell carcinoma (CESC)	Carcinoma	Squamous epithelium	3	305	up	0.25	0.35
cholangiocarcinoma (CHOL)	Carcinoma	mixed (glandular or squamous epithelium)	9	36	up	1.65E-12	4.73E-09
colon adenocarcinoma (COAD)	Carcinoma	glandular epithelium	41	286	up	1.62E-12	6.65E-04
esophageal carcinoma (ESCA)	Carcinoma	mixed (glandular or squamous epithelium)	11	184	up	3.67E-06	0.27
head and neck squamous cell carcinoma (HNSC)	Carcinoma	Squamous epithelium	44	520	up	1.62E-12	3.87E-07
kidney renal clear cell carcinoma (KIRC)	Carcinoma	glandular epithelium	72	533	up	1.62E-12	<1E-12
kidney renal papillary cell carcinoma (KIRP)	Carcinoma	glandular epithelium	32	290	up	<1E-12	2.44E-03
liver hepatocellular carcinoma (LIHC)	Carcinoma	Liver cell epithelium	50	371	up	1.62E-12	2.93-10
rectal adenocarcinoma (READ)	Carcinoma	glandular epithelium	10	166	up	0.02	0.11
pancreatic adenocarcinoma (PAAD)	Carcinoma	glandular epithelium	4	178	down	0.19	0.41
prostate adenocarcinoma (PRAD)	Carcinoma	glandular epithelium	52	497	down	8.09E-05	0.06
bladder urothelial carcinoma (BLCA)	Carcinoma	transitional epithelium	19	408	down	0.42	0.03
thyroid carcinoma (THCA)	Carcinoma	mixed	59	505	down	2.81E-10	3.44E-10
glioblastoma multiforme (GBM)	Brain and spinal cord cancers	brain and spinal cord	5	156	down	6.00E-02	0.99
Sarcoma	Sarcoma	connective tissues	2	260	up	0.49	1.62E-12
Thymoma	Lymphomas and myeloma	The lymphatic system (thymus)	2	120	down	0.89	0.03

*comparison of primary tumor tissue to normal tissue

#Classification based on https://www.cancerresearchuk.org/what-is-cancer/how-cancer-starts/types-of-cancer#carcinomas

Supplementary table 22. The studies used in the discovery dataset

					Subjects involving in this investigation				
Study Name	Institute	Country	study design	study period	Case No.	Control No.	Total No.	Criteria of selecting Participants	Ref (PMID)
The Alpha-Tocopherol; Beta-Carotene Cancer Prevention	National Cancer Institute	Finland	Cohort	1985-1993	1111	666	1777	The 50- to 69-year-old participants were randomized in a factorial design to take a pill daily for five to eight years that contained one of the following: 50 milligrams (mg) of dl-alpha-tocopheryl acetate, 20 mg of alltrans-beta-carotene, both, or a placebo.	8205268
The Vanderbilt Lung Cancer Study	Vanderbilt	USA	hospital CC*	2007-present	723	727	1450	Lung cancer cases were identified from the Vanderbilt tumor registry. Controls were randomly selected from BioVU participants, excluding cancer patients, and were matched to cases on age (+/- 5 years), sex, and race.	
Canadian screening study	University Health Network (UHN); British Columbia Construction Association (BCCA)	Canada	screening cohort	2004-2011; 2008-2013	212	443	655	The population-based sample included current and former smokers between 50 and 75 years of age without a history of lung cancer. Eligible participants had a 3-year risk of lung cancer of at least 2% as determined by a prototype of risk-prediction models in the Prostate, Lung, Colorectal, and Ovarian Cancer Screening Trial.	24004118
Cancer de Pulmon en Asturias	University of Oviedo	Spain	Hosp.CC*	2002-2012	716	686	1402	Lung cancer cases were incident cases of histologically confirmed lung cancer between 30 and 85 years of age and residents in the geographical area of each participating hospital. Controls were selected from patients admitted to those hospitals with diagnoses unrelated to the exposures of interest and individually matched by ethnicity, gender, age (± 5 years) and hospital.	23013535
The Carotene and Retinol Efficacy Trial	Fred Hutchinson Cancer Research Center	USA	Cohort	Recruitment 1985-1996.	151	133	284	CARET was initiated in 1983 with two pilot studies that tested two doses of β-carotene with or without vitamin A (retinol) in two high-risk populations: 816 men with substantial occupational exposures to asbestos and 1029 men and women who were either current or former cigarette smokers (<6 years since quitting) with a smoking history of at least 20 pack-years.	15572756
Copenhagen lung cancer study	Copenhagen	Denmark	Hosp.CC*	2004 (controls); 2012-2013 (cases)	583	1414	1997	Cases were diagnosed histologically with treatable lung cancer. Controls were participants of the Copenhagen General Population Study without lung cancer.	22441734
Environment and Genetics in Lung Cancer Study Etiology	National Cancer Institute	Italy	Pop.CC*	2002-2005	1803	1750	3553	Primary lung cancer cases were identified from 13 hospitals, which covered approximately 80% of incident lung cancer cases. Controls were randomly selected from the Lombardy Regional Health Service database and were frequency-matched to cases based on sex, 5-year age group, and area of residence.	18538025

German lung cancer study	Helmholtz Zentrum München (HMGU); German Cancer Research Center (DKFZ)	Germany	Mixed CC*	1998-2013	816	223	1039	The German Cancer Research Center (DKFZ) has recruited more than 1,000 lung cancer cases at and in collaboration with the Thoraxklinik Heidelberg.	18483334
Harvard Lung Cancer Study	Harvard/Mass General Hospital	USA	Hosp. CC	1992-2004	2133	528	2661	Cases included any person over the age of 18 years with a diagnosis of primary lung cancer that was further confirmed by an MGH lung pathologist. Controls were recruited from the friends or spouses of cancer patients or the friends or spouses of other surgery patients in the same hospital.	16214908
The IARC L2 Study	International Agency for Research on Cancer (IARC)	CE Europe	Pop./Hosp. CC*	2005-2013	1591	1892	3483	Cases were incident cancer patients collected from general hospitals in Russia, Poland, Serbia, Czech Republic, and Romania. Controls were recruited from individuals visiting general hospitals and out-patient clinics for disorders unrelated to lung cancer.	/
Kentucky Lung Cancer Research Initiative	Markey Cancer Center	USA	Pop. CC*	2012-2014	93	131	224	Cancer cases were recruited from the Kentucky Cancer Registry at the time of diagnosis and controls were from a random digit dialing process from the same region.	/
Liverpool Lung Cancer Project	The University of Liverpool	UK	Cohort	1999-2007; 1999-2011	116	181	297	The LLP has two components: 1) a case-controlled study of newly-diagnosed cases of lung cancer that will provide a baseline, risk assessment; and 2) a prospective cohort study to be carried out over a 10-yr period that will identify markers of preclinical carcinogenesis.	25873368
MD Anderson Cancer Center Study	MD Anderson Cancer Center	USA	Hosp. CC*	1994-current	971	954	1925	Cases were newly-diagnosed and histologically confirmed lung cancer patients recruited from UTMDACC. Controls were healthy individuals without a history of cancer.	17470739
The Malmö Diet and Cancer Study	Lund University	Sweden	Cohort	1991-1996	162	169	331	Subjects were aged 44 to 74 years of age and living in Malmö, Sweden.	11916347
Multiethnic Cohort Study	University of Hawaii	USA	Cohort	Recruitment 1993-1996	32	51	83	Subjects were aged 45-74 years at recruitment, primarily from five ethnic/racial groups – African Americans and Latinos and Japanese Americans, Native Hawaiians and whites.	15229477
New England Lung Cancer Study	Dartmouth College	USA	Pop. CC*	2005-2007	172	171	343	Cases were histologically confirmed primary incident lung cancer using the New Hampshire State Cancer Registry and the Dartmouth-Hitchcock Medical Center (DHMC) Tumor Registry. Control participants were identified using a commercial database and matched to lung cancer cases within 5-year age groups, sex and county.	20049123
Clalit National Israeli Cancer Control Center-lung cancer study	Carmel Medical Center & Technion	Israel/Europe	Pop.CC*	2005-ongoing	663	510	1173	This case-control study of newly diagnosed lung cancer cases of any histology and population age/sex/ethnicity-matched "healthy" controls	25924736
The Nijmegen Lung Cancer Study	Radboud University Medical Centre	The Netherlands	Pop. CC*	2002-2008	434	443	877	The Netherlands patients with lung cancer were identified through the population-based cancer registry of the Netherlands Comprehensive Cancer Organisation in Nijmegen, the Netherlands. The cancer-free controls were selected from participants of the “Nijmegen Biomedical Study”.	Control: 17568781; Case: 20418888

Norway Lung Cancer Study	National Institute of Occupational Health (NIOH)	Norway	Pop. CC*	1986-2005	323	417	740	Early-stage NSCLC cases and healthy controls at the time of enrollment were Caucasians of Norwegian origin and were recruited from Western Norway.	18258609
Northern Sweden Health and Disease Study	Umeå University	Sweden	Cohort	1985-present	237	236	473	All residents in the Västerbotten County were invited to participate by attending a health check-up at 40, 50 and 60 years of age.	14660243
The Prostate Lung Colorectal and Ovarian Cancer Screening Trial	National Cancer Institute	USA	Cohort	1992-2001	1183	718	1901	155,000 men and women age 55 to 74 years were recruited from 1992 to 20014.	22031728
ReSoLucent	University of Sheffield	UK	Mixed CC*	2005-2014	577	297	874	Lung cancer cases diagnosed at ages older than 60 years were recruited if they reported a family history of lung cancer. The matched controls were recruited through several major cancer treatment centres, mostly in North Trent.	/
Tampa Lung Cancer Study	Washington State University	USA	Hosp. CC*	1999-2003	101	146	247	All subjects were recruited from the H. Lee Moffitt Cancer Center (Tampa, FL). All cases were patients diagnosed with primary lung cancer and were identified between 1999 and 2002. All cases were diagnosed within one year prior to recruitment into the study and were histologically confirmed by the Pathology Department at the H. Lee Moffitt Cancer Center. We recruited control subjects at the Lifetime Cancer Screening Center affiliated with the H. Lee Moffitt Cancer Center. All control subjects were recruited after an initial verbal screening to determine that they had no previous diagnosis of cancer, and none of the controls recruited into this study were diagnosed with any form of cancer or premalignancy after screening. The eligible pool of control subjects was restricted to those individuals with the same age at diagnosis (± 5 years), race, and sex as the case subjects.	15901990
Total Lung Cancer: Molecular Epidemiology of Lung Cancer Survival	Moffitt Cancer Center; Tampa; FL	USA	case only	2012--present	427	0	427	458 lung cancer patients were recruited for Moffitt Cancer Center's Total Cancer Care™ protocol	23839018
Mount Sinai Hospital-Princess Margaret Hospital Study	Mount Sinai Hospital; Princess Margaret Hospital	Canada	Hosp. CC*	2008-2012	521	141	662	Lung cancer cases were recruited at the hospitals in the network of the University of Toronto. Controls were selected randomly from individuals registered in the family medicine clinics databases and were frequency matched with cases on age and sex.	24880342; 24947688

Supplementary table 23. The germline mutations affecting histologic specific lung cancer risk with high effect with association *P* value of less than 5.0 x 10^{-8} and OR value of more than 2.0 in the discovery dataset

				Discovery Dataset								Replication Dataset							
Histology of Ling cancer	CHR	SNP	BP*	A1	F_A[a]	F_U[b]	A2	OR (95% CI)			P-value	A1	F_A[a]	F_U[b]	A2	OR (95% CI)			P-value
Squamous cell carcinoma	22	rs150665432	45608215	A	0.014	0.0049	G	2.942	2.267	3.816	1.68E-17	A	0.004686	0.002997	G	1.566	0.7689	3.191	0.2127
Adenocarcinoma	1	rs61816761	152285861	G	0.003	0.0003	A	9.454	4.172	21.43	4.60E-11	A	0.01467	0.01815	G	0.8054	0.6048	1.073	0.138
Adenocarcinoma	11	rs56009889	108196896	T	0.006	0.0007	C	8.151	4.857	13.68	2.74E-21	T	0.004198	0.001403	C	3	1.511	5.958	0.000976
Adenocarcinoma	22	rs150665432	45608215	A	0.012	0.0049	G	2.469	1.946	3.134	1.49E-14	A	0.004895	0.002997	G	1.637	0.9427	2.841	0.07707
Small cell lung cancer	1	rs61816761	152285861	G	0.004	0.0003	A	14.2	5.662	35.62	8.71E-14	A	0.01515	0.01815	G	0.8322	0.4979	1.391	0.4829
Small cell lung cancer	3	3:9970073	9970073	G	0.006	0.0011	C	5.423	3.064	9.598	7.186E-11	0	0	0	C	NA	NA	NA	NA
Small cell lung cancer	6	rs17843743	32614774	I	0.003	0.0002	D	18.53	4.789	71.72	3.79E-09	I	0.4364	0.4138	D	1.097	0.9646	1.248	0.1583
Small cell lung cancer	22	rs150665432	45608215	A	0.015	0.0049	G	3.061	2.197	4.267	3.667E-12	A	0.004744	0.002997	G	1.586	0.6166	4.078	0.3345

*Physical positions are based on NCBI build 37 of the human genome.

[a]Frequency of this allele in cases (F_A)

[b]Frequency of this allele in controls (F_U)

Supplementary Table 24. Genotype frequencies based on the agreement between OncoArray genotyping and Affymetrix genotyping

	OncoArray Genotyping		
Affymetrix Genotyping	major/major	major/minor	minor/minor
major/major	a	b	c
major/minor	d	e	f
minor/minor	g	h	i
