# Supplementary material for: Protein-altering germline mutations implicate novel genes related to lung cancer development
Source: Nat Commun. 2020 May 11;11:2220. doi: 10.1038/s41467-020-15905-6 (PMC7214407; doi:10.1038/s41467-020-15905-6)
Supplement: Supplementary file 2 — Reporting Summary [file 41467_2020_15905_MOESM2_ESM.pdf]

nature research

Corresponding author(s): Christopher I. Amos and Xuemei Ji

Last updated by author(s): Nov 15, 2019

Reporting Summary

Nature Research wishes to improve the reproducibility of the work that we publish. This form provides structure for consistency and transparency in reporting. For further information on Nature Research policies, see Authors & Referees and the Editorial Policy Checklist .

Statistics

For all statistical analyses, confirm that the following items are present in the figure legend, table legend, main text, or Methods section.

n/a	Confirmed	
☐	☒	The exact sample size (n) for each experimental group/condition, given as a discrete number and unit of measurement
☐	☒	A statement on whether measurements were taken from distinct samples or whether the same sample was measured repeatedly
☐	☒	The statistical test(s) used AND whether they are one- or two-sided<br>*Only common tests should be described solely by name; describe more complex techniques in the Methods section.*
☐	☒	A description of all covariates tested
☐	☒	A description of any assumptions or corrections, such as tests of normality and adjustment for multiple comparisons
☐	☒	A full description of the statistical parameters including central tendency (e.g. means) or other basic estimates (e.g. regression coefficient) AND variation (e.g. standard deviation) or associated estimates of uncertainty (e.g. confidence intervals)
☐	☒	For null hypothesis testing, the test statistic (e.g. F, t, r) with confidence intervals, effect sizes, degrees of freedom and P value noted<br>*Give P values as exact values whenever suitable.*
☐	☒	For Bayesian analysis, information on the choice of priors and Markov chain Monte Carlo settings
☐	☒	For hierarchical and complex designs, identification of the appropriate level for tests and full reporting of outcomes
☐	☒	Estimates of effect sizes (e.g. Cohen's d, Pearson's r), indicating how they were calculated

Our web collection on statistics for biologists contains articles on many of the points above.

Software and code

Policy information about availability of computer code

Data collection	This item is not really applicable, but we did use the Oncoarray for genotyping which has been described previously (PMCID: PMC5224974 DOI:10.1158/1055-9965.EPI-16-0106)
Data analysis	PLINK version 1.9 was used for case-control association analyses (https://www.cog-genomics.org/plink2/ also at https://github.com/chrchang/plink-ng/tree/master/1.9). We performed meta-analyses with the application of R package 'meta' (http://www.imbi.uni-freiburg.de/lehre/lehrbuecher/meta-analysis-with-r). Principal component analysis was performed based on GWAS data with the EIGENSTRAT program for both discovery and replication datasets, respectively. We used the Statistical analysis software (SAS) version 9.3 for other analyses (http://support.sas.com/software/93/).

For manuscripts utilizing custom algorithms or software that are central to the research but not yet described in published literature, software must be made available to editors/reviewers. We strongly encourage code deposition in a community repository (e.g. GitHub). See the Nature Research guidelines for submitting code & software for further information.

Data

Policy information about availability of data

All manuscripts must include a data availability statement. This statement should provide the following information, where applicable:

- Accession codes, unique identifiers, or web links for publicly available datasets
- A list of figures that have associated raw data
- A description of any restrictions on data availability

The data that support the findings of this study are available. The access numbers are "phs001273" for Oncoarray study, "phs001681.v1.p1" for Affymetrix study, and "phs001783.v1.p1" and "phs001858.v1.p1" for MSK-IMPACT study in dbGAP.

Field-specific reporting

Please select the one below that is the best fit for your research. If you are not sure, read the appropriate sections before making your selection.

[x] Life sciences [ ] Behavioural & social sciences [ ] Ecological, evolutionary & environmental sciences

For a reference copy of the document with all sections, see nature.com/documents/nr-reporting-summary-flat.pdf

Life sciences study design

All studies must disclose on these points even when the disclosure is negative.

Sample size	The sample size comprised all available data for identifying and estimating protein altering variants conferring a high risk of lung cancer. Exome-wide association analyses were restricted to European-descent populations to avoid confounding between ethnicity and minor allele frequency that varies among populations. The discovery sample comprised 28,878 individuals and the validation set included 10,268 additional individuals. We utilized the germline and matched tumor data for 2,127 lung cancer patients of European ancestry in an additional, independent MSK-IMPACT dataset. A total of 7570 samples from The Cancer Genome Atlas (TCGA, https://tcgadata.nci.nih.gov/tcga/) level 3 RNA-seq data and clinical patient data related to 19 cancer types were used to investigate whether or not the expression of ATM or KIAA0930 were associated with the primary cancer. Harvard lung expression data, including a total of 203 samples, were included in the analysis.
Data exclusions	Only individuals with deduced European ancestry greater than 80% were retained in GWAS datasets. Samples with genotyping call rates of <95% were excluded. The OncoArray consortium participants who were lacking disease status (because they were not part of the lung cancer related studies), who were close relatives (second-degree relatives or closer) or who were duplicate individuals, or who had a low call rate of genotype data, or who did not pass quality control, or who were non-European, were excluded from the current study. There were 5742 participants in the OncoArray consortium who were also genotyped in the replication phase for investigating genotyping fidelity, and therefore these samples were also excluded from the analysis in the discovery phase. Of all subjects in the Affymetrix Axiome array study, the participants who were lacking disease status, or who were non-European, or whose samples did not pass quality control, were excluded. There are no data exclusions for TCGA data and Harvard lung expression data.
Replication	We used five methods to make sure that our findings are reliable. First, we discovered and verified the results in two independent datasets. Second, we used 20 gene expression datasets to confirm the involvement of KIAA0930 in cancer development. Third, we analyzed the data by ethno-geographic populations in the discovery dataset to investigate the significance and validation of our discovered mutations. Fourth, we confirmed the genotyping fidelity of the selected germline mutations in both datasets by repeating genotyping 5742 subjects of the replication phase along with the discovery phase. The results for rs56009889 and rs150665432 had excellent concordances confirmed their genotyping fidelity in both phases. Fifth, we validated the reliability of genotyping data of the mutations by comparing and confirming the agreement of their MAFs in unaffected individuals of our both datasets to those in public sequencing projects or datasets. Finally, the facts that all homozygotes of rs56009889 mutation had lung adenocarcinoma and all homozygotes of rs150665432 mutation had lung cancer supporting the association that these two mutations have in lung cancer etiology. The investigation of the structure-based prediction, loss of heterozygosity of ATM-L2307F, isoforms and genes expression level further supported the biological relevance of the novel mutations and their related genes.
Randomization	This is a case-control study and randomization is not possible. We adjusted for principal components to control for potential minor effects of allelic variation among European populations. We also performed stratified analyses to identify variations in effect sizes according to histology, smoking behavior, sex and age. We performed further sensitivity analyses to understand effects of age on genetic effects of risk for lung cancer.
Blinding	Genotyping on both arrays that were analyzed was performed blinded to the case or control status and all demographic factors.

Reporting for specific materials, systems and methods

We require information from authors about some types of materials, experimental systems and methods used in many studies. Here, indicate whether each material, system or method listed is relevant to your study. If you are not sure if a list item applies to your research, read the appropriate section before selecting a response.

Materials & experimental systems

n/a	Involved in the study	
[x]	[ ]	Antibodies
[x]	[ ]	Eukaryotic cell lines
[x]	[ ]	Palaeontology
[x]	[ ]	Animals and other organisms
[x]	[ ]	Human research participants
[x]	[ ]	Clinical data

Methods

n/a	Involved in the study	
[x]	[ ]	ChIP-seq
[x]	[ ]	Flow cytometry
[x]	[ ]	MRI-based neuroimaging
